# Bridging the SME reporting gap: A new model for predicting Scope 1 and 2 emissions

**DOI:** 10.1111/jiec.70106

**Published:** 2025-09-23

**Authors:** Alec Phillpotts, Anne Owen, Jonathan Norman, Anna Trendl, John Gathergood, Norbert Jobst, David Leake

**Affiliations:** 1 Sustainability Research Institute, University of Leeds, Leeds, UK; 2 Behavioural Science, Lloyds Banking Group, London, UK; 3 Warwick Business School, University of Warwick, Coventry, UK; 4 School of Economics, University of Nottingham, Nottingham, UK; 5 Portfolio Analytics, Lloyds Banking Group, London, UK

**Keywords:** emission modeling, financial transaction data, industrial ecology, SMEs, scope 1, scope 2

## Abstract

We present a novel statistical model for predicting Scope 1 and 2 emissions for small and medium-sized enterprises (SMEs). Trained on financial transaction data from over 100,000 UK SMEs, the model targets a business segment excluded from formal emissions reporting and often under-engaged in sustainability efforts. By leveraging scalable, objective data, our approach offers an accessible alternative to existing methods that rely on either coarse sectoral averages or detailed, resource-intensive firm-level activity data. In developing the model, we evaluate a range of predictors and find that incorporating industry-level variables beyond basic emission intensity significantly enhances predictive accuracy. We also observe diminishing returns from additional model complexity, reinforcing the value of a parsimonious, low-input design. The final model achieves RSQ values of 0.89 for Scope 1 and 0.72 for Scope 2, improves accuracy by up to 50% compared to sector-level estimates, and performs reliably on out-of-sample data. Our findings provide a simpler approach to emissions estimation for SMEs, supporting broader climate engagement among smaller actors.

**Supplementary Information:**

The online version of this article (doi:10.1111/jiec.70106) contains supplementary material, which is available to authorized users.

## INTRODUCTION

Although small and medium-sized enterprises (SMEs) constitute 99% of the United Kingdom's business population (Department for Business, Energy & Industrial Strategy (BEIS), 2022), their environmental impact remains insufficiently understood. Unlike larger firms, SMEs are exempt from formal emissions reporting requirements under frameworks such as the United Kingdom's Streamlined Energy and Carbon Reporting (SECR) scheme, which mandates the disclosure of emissions as part of broader extra-financial reporting^1^ (HM Government, 2019; Meath et al., 2016). Globally, frameworks like SECR have increased the number of companies measuring and reporting their emissions (Assael et al., 2023; Tomar, 2023); however, the focus on large enterprises has led to a situation where only a subset of firms consistently report their emissions (Nguyen et al., 2021).

Extending reporting obligations to SMEs presents a complex challenge. While SMEs have a significant environmental impact collectively, their individual contributions are often minimal. Moreover, SMEs face numerous internal and external barriers to enhancing their sustainability, many of which are unique to firms of their size (Alipour & Rahimpour, 2020; Normative, 2024; Oyewole et al., 2024). Among these, information barriers and the inability to measure and monitor emissions are frequently cited as key challenges (British Business Bank (BBB), 2024). The process of emission measurement involves gathering detailed data on industrial processes and technologies from multiple sources, tracking annual consumption of fuel and energy in physical units, and applying calculations using relevant conversion factors. This is a challenging, laborious task, even for dedicated teams within big corporations (Assael et al., 2023; Goldhammer et al., 2017). These barriers, combined with regulatory exemptions, have allowed SMEs to remain largely under-engaged on the topic of sustainability (BBB, 2024).

To bridge the SME reporting gap, we develop a prediction model using a series of hierarchical regressions to estimate Scope 1 and 2 emissions requiring only minimal, widely available inputs: company turnover, industry classification, and industry-level characteristics drawn from public accounts. To tailor our model to the SME use case, it is fitted to emission estimates derived from anonymized financial transaction data (FTD) for over 100,000 UK SMEs.

By relying on routinely collected FTD rather than detailed activity data, this model places fewer requirements on SMEs while still capturing the key drivers of Scope 1 and 2 emissions. FTD-based emission estimates capture a simplified version of emissions, and their validation against actual emissions is challenging due to the scarcity of alternative data. Here, we argue that, for SMEs, precision is not paramount. A degree of absolute accuracy can be reasonably traded for substantial gains in efficiency and scalability. Prior work confirms FTD as a credible alternative to survey-based emissions data (Trendl et al., 2023), noting key advantages such as reduced calculation effort, scalability, and real-time application (Wells et al., 2025). Moreover, using a standardized, transparent methodology across firms helps avoid the inconsistencies associated with error-prone self-reported data (Financial Reporting Council, 2021).

Through this work, we contribute to the understanding of emissions within this critical segment of the economy and provide a simple methodology to underpin more practical and accessible solutions for SMEs' emission estimation. We argue that developing this model into a publicly accessible tool would enable SMEs and other stakeholders to generate reliable emissions estimates, while encouraging greater engagement with sustainability among currently disengaged smaller actors.

The remainder of this article is organized as follows. First, we provide an overview of emission accounting for firms, the stakeholders of SME emissions data, and the current approaches for estimating non-reported emissions. Following this, we introduce the materials and methods used to calculate Scope 1 and 2 emissions from FTD and the statistical techniques used in developing our prediction model. Subsequently, we present the results of our model, along with robustness checks and out-of-sample performance evaluations. The article concludes with a discussion of our key findings, their practical implications, limitations, and suggestions for future research directions.

## BACKGROUND AND CONTEXT

### Defining emission scopes

A firm's carbon footprint refers to the greenhouse gases (GHGs) that are produced as a by-product of its economic activities. The concept is defined by the accounting principles, system boundaries, reporting standards, and framework for calculations laid out in the GHG Protocol Corporate Accounting and Reporting Standards (WRI & WBCSD, 2004). This framework outlines clear, differentiated “Scopes” that make up a firm's footprint:

- Scope 1—Direct GHG emissions from operations that are owned or controlled by the company. This includes stationary combustion, mobile combustion, and process emissions.
- Scope 2—Indirect GHG emissions from the generation of purchased electricity, steam, and heating/cooling consumed by the company.
- Scope 3—Other indirect GHG emissions from the upstream and downstream value chain.

These definitions indicate that one firm's Scope 2 and 3 emissions are the allocated Scope 1 emissions of another, emphasizing that purchasers should account for emissions driven by their demand and highlighting the need for broader emission reduction strategies beyond their own production (Hertwich & Wood, 2018).

Emission reporting regulations, such as SECR, have historically prioritized the mandatory disclosure of energy-related Scope 1 and 2 emissions, with Scope 3 emissions often considered ambiguous, voluntary, and deferred for future consideration (Assael et al., 2023; Hansen et al., 2022; Hettler & Graf-Vlachy, 2024; Nguyen et al., 2021). The focus on Scopes 1 and 2 facilitates manageable measurement, but provides a limited view, as Scope 3 can make up the majority of a firm's emission profile (Harangozo & Szigeti, 2017; Hertwich & Wood, 2018; Matthews et al., 2008; Stein & Khare, 2009). Given the distinct scopes, here we estimate Scope 1 and 2 emissions independently, noting the additional theoretical considerations for Scope 3 as well as the opportunity and novelty involved in leveraging FTD as a record of upstream emissions.

### Implications of the SME reporting gap

Emission measurement is widely recognized as a critical first step for businesses in advancing sustainability, grounded in the principle “what gets measured gets managed” (WRI & WBCSD, 2004, p. 11). Research supports this, showing that firm emission measurement and disclosure is often followed by reductions (Tomar, 2023), through the creation of emission baselines and an improved understanding of emission sources (Harangozo & Szigeti, 2017; Talbot & Boiral, 2013). In the absence of this information, SMEs remain limited in their ability to identify and implement effective decarbonization strategies. The lack of emissions data also places SMEs at a competitive disadvantage with both downstream consumers (Deloitte, 2023; McKinsey, 2023) and upstream suppliers (Hettler & Graf-Vlachy, 2024; World Economic Forum, 2022) increasingly focusing on environmental factors in decision-making. Firms with clearly defined sustainability strategies and emission measurement approaches may be favored over those without, leading to important long-term effects on company performance.

Additionally, policymakers and financial institutions (FIs) are impacted by the absence of comprehensive SME emissions data. For the policymaker, increased access to and a greater understanding of SME emissions data are essential for developing effective environmental policies capable of driving sector-wide behavioral and operational change. This presents a significant challenge to realizing the United Kingdom's legally binding Carbon Budgets, which are designed to provide a structured and measurable pathway to achieving net-zero GHG emissions by 2050 (Department for Business, Energy & Industrial Strategy, 2021). For FIs, these data gaps make it difficult to design, assess, and implement financed emissions strategies, limiting their ability to align lending and investment portfolios with climate targets. Under the Partnership for Carbon Accounting Financials (PCAF, 2022) framework, FIs must measure and disclose their financed emissions. Without accurate SME emission data, FIs rely on estimation techniques, which introduce uncertainty and reduce the precision of portfolio-level climate impact assessments. Improving the quality and accessibility of SME emissions data would enable financial institutions to pursue more targeted green financing, with important knock-on effects for the wider economy (The Organisation for Economic Co-operation & Development (OECD), 2024).

### Methods for estimating non-reported emissions

In the absence of reported emissions and internal physical usage data, a common approach for predicting firm-level emissions involves combining industry-average emission intensity factors, derived from official statistical data or established environmentally extended input–output tables, with firm-specific variables such as revenue and employment (Hirvonen et al., 2021; OECD, 2023). This methodology is employed in both financed emission calculation (PCAF, 2022) and in broader assessments of the overall contribution of SMEs emissions. Such estimates suggest that SMEs may account for approximately half of all UK business-driven emissions (BBB, 2021, p. 15).

While straightforward and simple to produce, sole reliance on industry emission intensity factors ignores firm-level heterogeneities within an industry and assumes a constant relationship between emissions and the specified economic variable (European Commission, 2022). Additionally, industry averages are skewed by larger firms and may not represent smaller businesses. Despite their physical numbers, SMEs contribute far less to total industry turnover, labor, and emissions.

Beyond basic estimates, more sophisticated models have been developed using machine learning and statistical methods, summarized in Table 1 (Assael et al., 2023; Goldhammer et al., 2017; Griffin et al., 2017; Heurtebize et al., 2022; Nguyen et al., 2021). These models rely on publicly disclosed firm-level emission data, resulting in their development being predominantly based on larger firms. This limits applicability to smaller firms (Assael et al., 2023) and can introduce inconsistencies due to quality and reliability issues of self-reported data (Dragomir, 2012; Talbot & Boiral, 2013). Moreover, the reliance on self-reported data places an important limitation on model sample sizes, with the largest study involving 4360 firm-year observations (Assael et al., 2023).

**TABLE 1:** Summary of existing prediction models for company emissions.

Model	Observations	Sample data	Model	Scope 1 and 2 treatment
Goldhammer et al. (2017)	93	Self-reported figures	Ordinary least squares	1 and 2 jointly
Griffin et al. (2017)	1657		Ordinary least squares	1 and 2 jointly
Nguyen et al. (2021)	2289		Machine learning	1 and 2 individually
Heurtebize et al. (2022)	3500		Machine learning	1 and 2 individually
Assael et al. (2023)	4360		Machine learning	1 and 2 individually

## MATERIALS AND METHODS

The prediction model presented here is developed using the anonymized FTD of businesses banking with Lloyds Banking Group (LBG), one of the United Kingdom's largest banks, with a business banking client base of over 900,000 firms (Lloyds Banking Group, 2023, p. 12). All businesses included in our analysis are SMEs, henceforth defined as firms with an annual turnover of less than £36 million. The model is based on the single year 2021, given the availability of both internal banking data and official industry data. The intention of this paper is to serve as a case study, under the understanding that additional model years would rely on annual data updates. These model parameters would be expected to change only gradually over time, supporting most practical applications.

**FIGURE 1:**
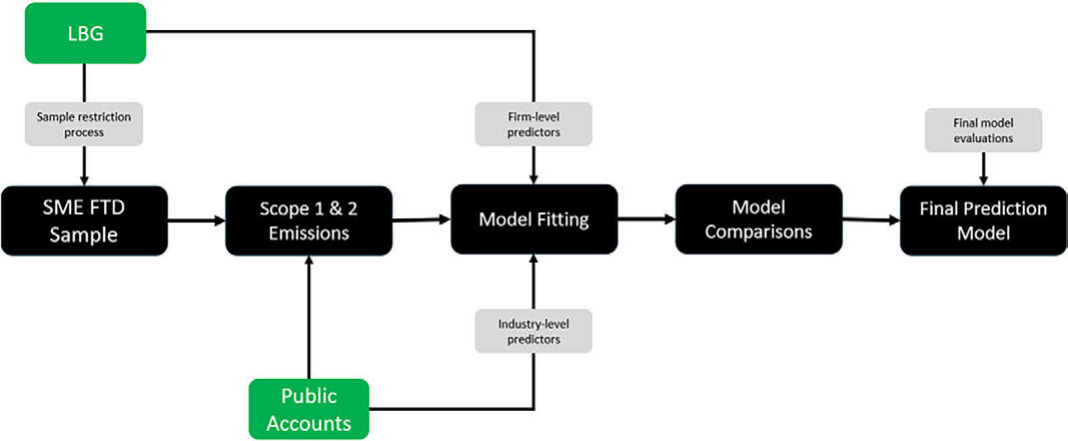
Overview of prediction model development. Black rectangles indicate the key stages of the modeling process, green denotes data sources, and gray highlights the specific inputs and processes required at each step. LBG, Lloyds Banking Group; SME, small and medium-sized enterprise.

The methodological approach to developing our model is described in the following subsections, while Figure 1 provides a schematic overview of this process. First, SME FTD is obtained from LBG, and an iterative sample restriction process is implemented. The FTD of the resulting sample is then combined with spend-based emission conversion factors, sourced from official UK government datasets, to estimate Scope 1 and 2 emissions. Starting with the most basic specification and increasing in complexity, we then fit a series of hierarchical regression models to the FTD emission estimates. For this, firm-level predictors are sourced from LBG, while industry-level predictors are drawn from official UK sources, including government databases and the Office for National Statistics (ONS). These models are evaluated and compared against each other for model performance. Our preferred model is selected, and further model evaluation testing is conducted.

### Sample selection

To ensure the analysis is based on high-quality and comprehensive financial data histories, a series of emission scope–specific restrictions is required. A full account of this process, including rationale and impact of each step, is provided within Supporting Information S1. These steps are designed to isolate SMEs who use LBG as their primary banking provider, excluding those with significant financial activities through other institutions.

Additionally, it is necessary to restrict our sample to SMEs for which financial data can reasonably proxy both firm revenue and emission-generating activities. As a result, we exclude agricultural SMEs due to their substantial process-related emissions not directly reflected in FTD. We also omit certain service-sector SMEs, such as those in insurance, real estate, and legal or accounting services, whose frequent handling of non-turnover credit lines (e.g., deposits) leads to systematic overestimation of turnover. Table 7 of Supporting Information S1 provides a full list of explanations of exclusions where relevant, along with sample observations for each industry.

With the sample restrictions steps in place, our final sample for Scope 1 consists of 39,704 companies across 44 industries. Our Scope 2 sample is larger, covering 92,714 companies across 54 industries, as restrictions are based solely on electricity spend, while Scope 1 restrictions span multiple spend categories. Collectively, these companies account for an estimated £70 billion in annual revenue and encompass over 95 million financial transactions in 2021.

### FTD-based emission estimates

#### FTD classification and adjustments

A transaction is defined as any spend that occurs on a business account or credit card, including electronic transfers, online transactions, and card payments. Each transaction is classified according to the bank's internal transaction classification system, which assigns recipient-based categories (Trendl et al., 2023; Wells et al., 2025). Of these categories, two are relevant for the estimation of Scope 1 and 2 emissions: “Energy and Utilities” and “Vehicle Fuelling.” The former refers to four sub-categories of expenditure, containing energy providers selling electricity, natural gas, and heating oil, while the latter refers to diesel and petrol purchased for mobile combustion. With these categories, we capture the Scope 1 and 2 emissions generated from a firm's energy use, mirroring what is required of larger firms under the SECR framework.

After categorization, we apply two additional adjustments to the spending amounts classified under “Energy and Utilities.” First, in instances where UK utility firms are known to provide electricity, gas, and heating oil to consumers, we disaggregate spending into Scope 1 and Scope 2 using industry-level averages on energy spend by energy type (ONS, 2021). Second, due to energy tariff bands, businesses consuming more energy typically pay less on a per-unit basis. We therefore use UK statistics on consumption bands and energy unit prices (DESNZ, 2024) to calculate adjustment factors for different consumption bands. We provide our methodology for these adjustments in Supporting Information S2.

#### Scope 1 and 2 emission conversion factors

Next, we create a set of conversion factors to capture the emissions associated with a unit purchase of different energy and fuel types. We divide emission per unit figures (CO_2_e/kwh (or L); BEIS & DEFRA, 2022) by average price paid by non-domestic consumers per unit (£/kwh (or L); DESNZ, 2024) to return emissions produced per pound spent (CO_2_e/£).

As spending within “Vehicle Fuelling” reflects the consumption of both petrol and diesel, we produce a single conversion factor through a weighted average based on the United Kingdom's consumption split between petrol and diesel (DESNZ & BIES, 2022) and the unit price difference. For gas and oil, we use an average conversion factor given the near-zero difference in individual factors. Finally, for electricity, our emission conversion factor is based on the UK grid's emission intensity (location-based method). These conversion factors are multiplied by the annual expenditures of our sample to estimate Scope 1 and 2 emissions based on their energy consumption. Tables containing direct emission conversion factor calculation and subsequent sample emission summary statistics can be found in Supporting Information S3.

### Hierarchical regression models

Given the nature of the data, where firms are nested within industries, we specify a hierarchical regression. This allows for industry-specific effects to vary while including non-independent observations within the same industry (Gelman & Hill, 2006).

To support the statistical reasoning of our model, we ensure a minimum industry sample size of 50 and adequate representation across turnover brackets, requiring at least 5 firms in each: below £250,000, £250,000–£1 million, and above £1 million. While there is no defined minimum number of observations required in each group to perform a hierarchical regression (Gelman & Hill, 2006), this is relatively close to the 50/50 rule, where a minimum of 50 groups and 50 units per group assists the hierarchical method of estimation (Ali et al., 2019).

#### Dependent variable and predictor variables

Following Goldhammer et al. (2017), we predict the natural logarithm (ln) of the firm's Scope 1 and 2 emissions. We apply the same transformation to all independent variables, allowing for coefficients to be interpreted as elasticities (the percentage change in the dependent variable for a one percent change in the independent variable). Table 2 introduces the predictor variables, providing the rationale for their inclusion and the method of procurement.

**TABLE 2:** Predictor variables for the hierarchical regression model.

Variable	Unit	Description	Calculation / Procurement
Industry-level			
Industry intensity	**kg Co**_**2**_ **e/£**	Representing the emissions produced per unit of output across each industry, this is the only industry variable to change to reflect the scope of emissions in focus. Currently, industry intensity is used exclusively in non-reported emissions estimation methods, as it serves as a key indicator of variation in emissions-generating activity across industries. For this reason, it is an important feature in our model.	This calculation utilizes the 2021 UKMRIO model (Owen & Kilian, 2025), a model of the UK economy designed to study the UK economy and its environmental impact. For Scope 1 emissions, the calculation involves simply dividing industry direct emissions (*F*) by industry output in purchasers’ prices (*X*). For Scope 2 emissions, we follow an approach outlined by Hertwich and Wood (2018) to return industry scope 2 emissions, which are divided by industry output in purchasers' prices (*X*).
Industry turnover skew	**%**	A variable capturing the share of industry turnover from large firms assesses industry skew. We interact this variable with industry intensity, under the hypothesis that where industry skew is high, industry intensity is heavily influenced by larger firms in the industry, potentially reducing the suitability of industry averages for SME. As a result, we interact skew with industry intensity in our models.	ONS industry data are used to generate a percentage figure for the share of turnover produced by firms with an annual turnover exceeding £1 million (ONS, 2024b).
Firm-level			
SIC Code	**SIC**	The Standard Industrial Classification (SIC) identifies a firm's primary business activities. Including an industry identifier allows for more accurate comparisons of expected emission intensity among firms performing similar activities.	SIC classifications are the standard industry categories in the United Kingdom, with official economic and environmental data commonly published on a two-digit SIC code basis, while it is standard practice for firms banking with LBG to be assigned SIC codes at both the two- and three-digit level by relationship managers. Of the 88 possible two-digit SIC codes (See Supporting Information S1), 44 are included in the Scope 1 sample and 54 in the Scope 2 sample.
Turnover	**£**	Turnover serves as an indicator of a firm's operational scale and is included in the analysis because, all else being equal, higher turnover typically correlates with greater emissions. However, firms with higher turnover may also benefit from economies of scale or possess greater capacity to invest in emission-reduction technologies, thereby decreasing their emission intensity.	Credit turnover is derived by summing the credit transactions recorded within a firm's bank account in the year.
Margin	**%**	Firm margin is included as firms operating at a greater financial margin may have the capacity to allocate resources toward sustainable practices, potentially lowering their emission intensity.	We calculate profitability by simply subtracting total debit from total credit, and make the variable relative by dividing the result by credit turnover.
Capital spend—assets	**%**	Capital expenditure reflects a firm's investment in assets. This variable is included because high capital spending on newer energy-efficient technologies may serve to lower a firm's emission intensity. For ease of calculation, we define capital expenditure as purchases that require financing.	Asset capital spend is calculated by summing the percentage of total spend, spent on asset financing and plant hire for the 3-year period ending 2021. We take an average of these percentages to gain a single figure, capturing asset spending across the 3-year window.
Capital spend—vehicles	**%**		Vehicle capital spend is calculated by summing the percentage of total spend, spent on vehicle financing for the 3-year period ending 2021. As above, we calculate a single figure, capturing vehicle spending across the 3-year window.

#### Model evaluation

We develop four progressively complex models to estimate firm-level emissions for each emissions scope, under the following hierarchical regression structure.

1 $${\rm{ln}}\;\;{Y_i} = {\beta _n}\;\left( {{\rm{fixed}}\_{\rm{effects}}} \right) + {\alpha _n}\left( {{\rm{variable}}\_{\rm{effects}}} \right) + {\varepsilon _i}$$

where:

- $\;{Y_i}$ represents the absolute emissions of firm *i*;
- ${\beta _n}$ represents the coefficients for fixed predictors whose effects do not vary across industries;
- ${\alpha _n}$ represents the coefficients for the variable predictors whose effects do vary across industries;
- ε represents the residual error term.

We examine predictor influence sequentially, starting with two variables and increasing to eight, evaluating the required complexity to explain the dependent variables (Cohen et al., 2013). The baseline model includes only industry classification and turnover. Building on this, we add industry-average emissions intensity to align with data used under PCAF, followed by an industry skewness variable to test whether the industry-average provides sufficient sectoral detail. Model 4 then introduces additional firm-level variables to capture intra-industry heterogeneity.

For each model, we present coefficients and statistical significance, indicating the strength and direction of predictor relationships to the dependent variables, along with their standard errors, reflecting the estimate uncertainties. We evaluate both model fit (R-squared - RSQ) and model fit with respect to complexity (Akaike information criterion - AIC).

Following model comparison, our preferred model is subjected to further rounds of evaluation. Predictive accuracy is evaluated against the PCAF framework, which assumes uniform firm-level emissions intensity within industries. Comparing predicted intensity (predicted emissions divided by turnover) with PCAF estimates provides a reference point for assessing prediction error, while identifying areas of model strength and weakness. We then test for stability and out-of-sample performance through an 80/20 train-test split with stratified sampling, applying a fivefold cross-validation (James et al., 2021) to ensure balanced representation and robust performance evaluation. A further round of out-of-sample testing is conducted on a sample of 50 large businesses that report under SECR. As SMEs are not required to disclose emissions, model performance can only be assessed against larger firms for external one-to-one validation. In the SECR sample, annual turnover ranges from £38 million to £211 million. By contrast, our model is fitted exclusively to emissions estimates for SMEs with turnover below £36 million, with 80% falling under £1 million. We also identified several inconsistencies and errors in the self-reported SECR data, introducing further uncertainty into this approach. Finally, we perform a sensitivity analysis to evaluate the robustness of results under varying sample selection criteria.

## RESULTS

### Model performance comparison

#### Scope 1

Table 3 presents summary statistics and coefficient estimates for each model iteration. Across all four model specifications, firm turnover consistently shows a strong, positive relationship with Scope 1 emissions. A 1% increase in turnover corresponds to an average of at least a 0.77% rise in Scope 1 emissions, with the effect strengthening in more complex models.

**TABLE 3:** Model summary.

	Scope 1								Scope 2							
	Model 1		Model 2		Model 3		Model 4		Model 1		Model 2		Model 3		Model 4	
	Coef	Std	Coef	Std	Coef	Std	Coef	Std	Coef	Std	Coef	Std	Coef	Std	Coef	Std
ln turnover	0.79***	0.01	0.77***	0.01	0.77***	0.02	0.79***	0.02	0.70***	0.01	0.77***	0.01	0.78***	0.01	0.79***	0.01
ln scp1_intensity			−0.08***	0.01	0.84***	0.21	1.26***	0.21								
ln scp2_intensity											0.16***	0.00	−1.49***	0.10	−1.28***	0.10
ln skew					−0.47***	0.03	−1.03***	0.04					0.01	0.03	−0.51***	0.04
intensity*skew					−0.34***	0.05	−0.42***	0.05					0.38***	0.03	0.34***	0.03
ln assets							0.02***	0.00							0.02***	0.00
ln vehicles							0.01***	0.00							−0.02***	0.00
ln margin							0.57***	0.02							0.55***	0.01
RSQ (fixed)	0.655		0.609		0.408		0.425		0.567		0.630		0.586		0.585	
RSQ (total)	0.798		0.801		0.891		0.882		0.690		0.721		0.717		0.728	
AIC	79,427		79,341		79,111		78,299		211,955		210,662		210,362		208,299	

Asterisks indicate significance level where *p* values < 0.05 = *, <0.01 = **, and <0.001 = ***.

In Model 2, we find that the inclusion of industry average only slightly improves model performance (an RSQ increase of only 0.003), with its coefficient being relatively weak and negative. Accounting for industry skew in Model 3 shifts the industry intensity coefficient to positive, and increases its magnitude. A 1% increase in industry intensity is now related to a 0.84% increase in Scope 1 emissions. The interaction term between industry intensity and industry skew suggests that for industries involving larger, dominant firms, industry intensity has a weaker effect on SME emissions. This supports the hypothesis that industry-wide emissions metrics may not fully capture SME emissions patterns. The 9% increase in model performance here highlights the importance of incorporating additional industry structure variables, with larger firms appearing to emit less per unit of turnover, which may be explained by an economies-of-scale effect.

Adding firm-specific factors beyond turnover does not significantly enhance model performance. While industry variables gain strength, firm-level predictors yield mixed results: A 1% increase in assets or vehicle spending leads to only a 0.02% and 0.01% rise in Scope 1 emissions, respectively. In contrast, a 1% increase in firm margin results in a notable 0.57% emissions increase, suggesting more profitable firms emit more, ceteris paribus. Despite these additions, model performance slightly declines (RSQ = 0.882).

#### Scope 2

In all Scope 2 models displayed in Table 3, turnover remains a strong predictor of emissions. While the initial strength of this variable is smaller for Scope 2, this coefficient increases to 0.79 with the final model.

Notably, we find the initial impact of industry intensity to be 0.16, significantly different from the relationship observed among Scope 1 emissions. The inclusion of industry skew in Model 3 again reverses the industry intensity coefficient, this time to negative, suggesting that firms in industries with a higher Scope 2 intensity have lower individual Scope 2 emissions, holding all else constant, with the effect diminishing as industry skew increases. This result may be driven by the calculation method of industry Scope 2 emissions, which is based on electricity expenditures from supply and use tables. Firms in industries with high electricity consumption generally secure lower unit prices, with larger firms benefiting most. This potential underestimation of total industry emissions may create a discrepancy between industry-wide and firm-level Scope 2 emissions. While the negative, statistically significant interaction term suggests an explanation centered on the influence of industry skew, other contributing factors could include regional tariff variations or industry-specific differences in electricity consumption patterns.

In Model 4, additional firm-level predictors improve RSQ to 0.728 and reduce AIC to 208,299. As in Scope 1, margin has the largest impact, while our capital assets variable has minimal influence (coefficient = 0.02). Interestingly, vehicle spending has a negative coefficient, where a 1% increase is associated with a 0.02% decrease in Scope 2 emissions.

#### Prediction model selection

**FIGURE 2:**
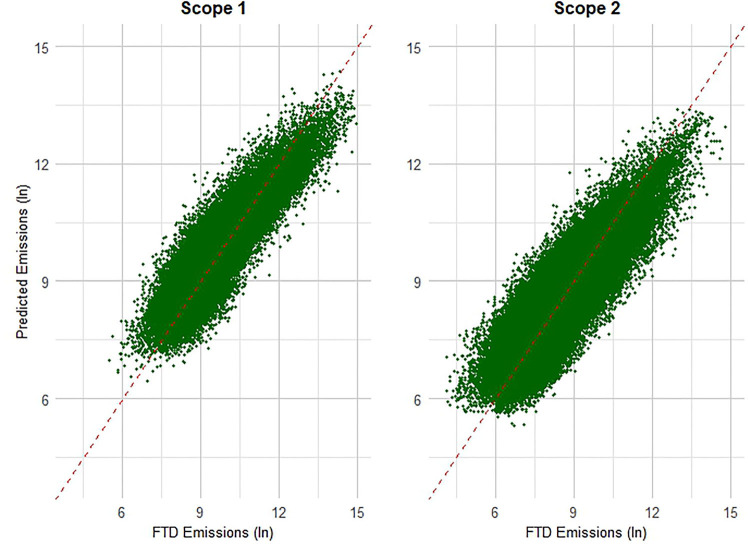
Model 3 predicted emissions against financial transaction data (FTD) emissions. Underlying data for this figure can be found in Table 1 of Supporting Information S5.

The preceding results indicate that the inclusion of additional firm-level variables did not significantly enhance model performance, despite the increased complexity and data resources needed to acquire these predictors. This indicates diminishing returns to model complexity; a simple, parsimonious solution can reach useful levels of predictive power while being practical to apply. Based on performance and applicability, we select Model 3 as our preferred model for each scope. Figure 2 presents the model prediction versus FTD emission estimates.

### Prediction model evaluation

To evaluate accuracy, we calculate the absolute error (AE) and absolute percentage error (APE) of Model 3's predictions relative to their FTD counterparts. Given the left-skewed distribution of firm sizes in the sample due to greater physical numbers of smaller firms, we report both the mean and median values of AE and APE.

#### PCAF comparison

Assessing differences in AE and APE between our model and the PCAF framework reveals large error reductions for Scope 1 emissions. Here, the PCAF framework results in a mean AE of 0.108 kg CO_2_e/£, while Model 3 improves this significantly, reducing the error to 0.047 kg CO_2_e/£, a reduction of over 50%. The decrease in median AE is smaller in absolute terms, but falling from 0.092 to 0.033 kg CO_2_e/£ represents another reduction of over 50%. For Scope 2 emissions, the reduction is more modest, with a decrease in both mean AE (0.033 to 0.022 kg CO_2_e/£) and median AE (0.022 to 0.014 kg CO_2_e/£).

**FIGURE 3:**
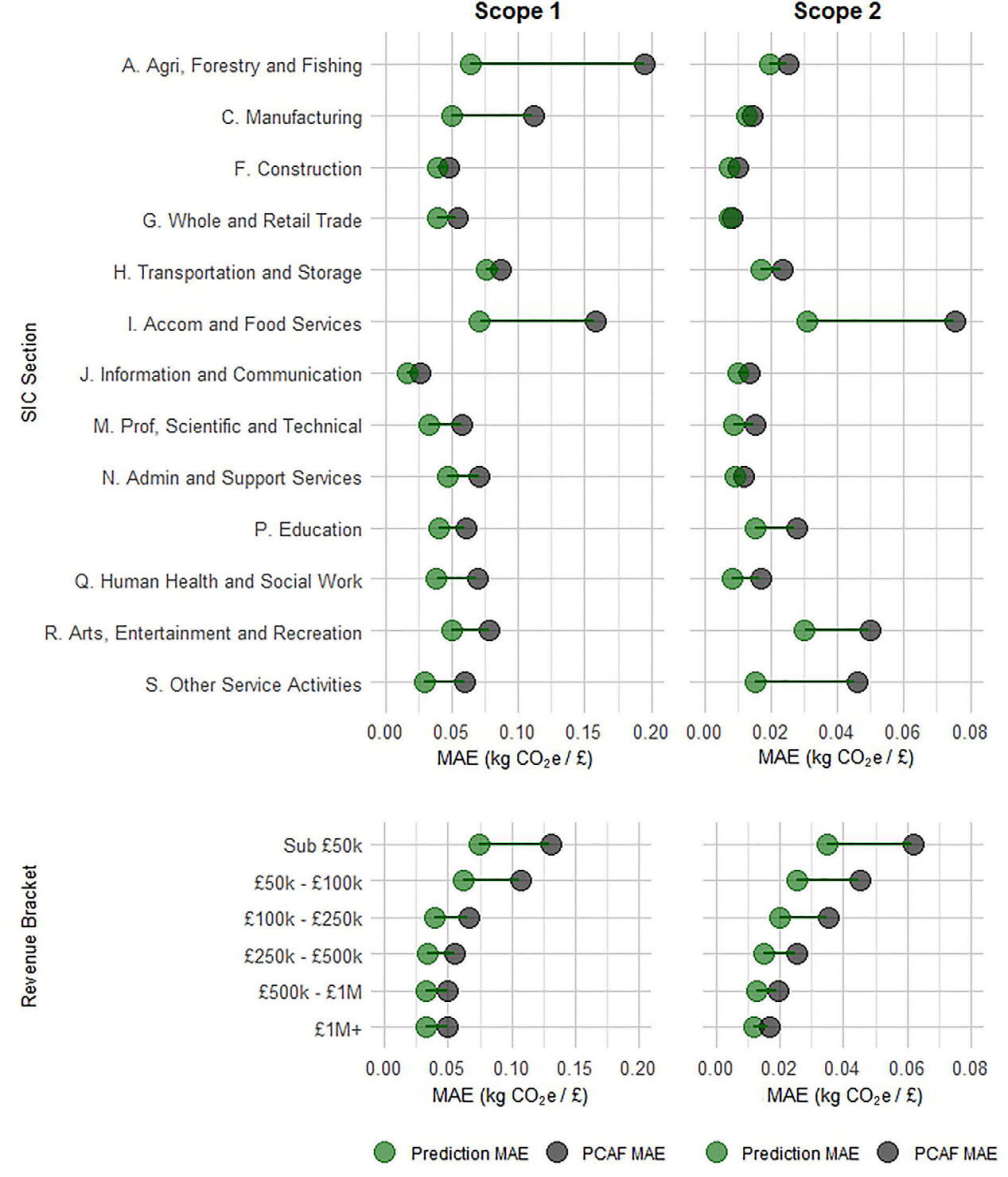
Comparison of mean absolute error (MAE) from Model 3 and Partnership for Carbon Accounting Financials (PCAF) across industry sectors and turnover brackets. Underlying data for this figure can be found in Table 2 of Supporting Information S5. PCAF, Partnership for Carbon Accounting Financials; SIC, Standard Industrial Classification.

We plot both PCAF and predicted mean AE by aggregated SIC section and turnover brackets in Figure 3. We observe that the greatest reduction in error occurs in industries with the largest starting error. However, error reductions are observed across all industries. Assessing the mean AE by firm size demonstrates that initial errors decrease with firm size and that predicted errors diminish consistently across all revenue categories. This highlights the model's effectiveness, significantly reducing the errors for smaller firms, which typically experience the highest errors under the average approach.

#### Out-of-sample performance

Table 4 presents the output from the fivefold cross-validation for Model 3. For each fold, we report the mean and median AE and APE, as well as the RSQ of the test data. The model consistently produces similar results across the folds, indicating stability. For Scope 1, the median AE across all folds was 6.8 tCO_2_e (std. = 0.03 tCO_2_e), and the average RSQ was 0.78 (s.d. = 0.00) for Scope 1. For Scope 2, the median AE across all folds was 2.4 tCO_2_e (s.d. = 0.01 tCO_2_e), with an average RSQ of 0.73 (s.d. = 0.00). This indicates that the model performs robustly when used to predict out-of-sample FTD-based emission estimates.

**TABLE 4:** Performance metrics for Model 3 test and train iterations.

	Scope 1					Scope 2				
		Mean		Median			Mean		Median	
Fold	RSQ	AE	APE	AE	APE	RSQ	AE	APE	AE	APE
		tCO_2_e	%	tCO_2_e	%		tCO_2_e	%	tCO_2_e	%
1	0.778	29.7	63.24	6.9	41.40	0.730	9.2	73.50	2.4	48.74
2	0. 778	31.0	63.35	6.8	41.45	0.730	9.6	74.65	2.4	48.69
3	0.782	30.5	62.57	6.8	41.35	0.730	9.4	73.96	2.4	48.21
4	0.782	30.9	62.13	6.9	41.12	0.729	9.2	73.95	2.4	48.53
5	0.776	30.4	62.75	6.9	41.26	0.728	9.7	74.22	2.4	48.79
**Mean**	**0.779**	**30.5**	**62.81**	**6.8**	**41.31**	**0.729**	**9.4**	**74.05**	**2.4**	**48.59**
**Std**	**0.003**	**0.5**	**0.50**	**0.03**	**0.13**	**0.000**	**0.2**	**0.42**	**0.01**	**0.23**

Abbreviations: AE, absolute error; APE, absolute percentage error.

Further out-of-sample testing is presented in Supporting Information S4, where we apply our models to predict emissions for 50 large businesses reporting under the SECR framework. We observed a median APE of 25.32% for Scope 1 emissions. Errors are substantially higher for Scope 2 emissions, with a median APE of 65.08%. Although these results are broadly in line with error levels exhibited in Table 4, we observe low RSQ scores and high mean errors, largely driven by a small number of extreme outliers.

#### Impact of sample selection

Up to this point, the results presented are based on the restriction steps laid out in Supporting Information S1. For transparency, in Table 5, we now present a sensitivity analysis on Model 3's performance as we alter trimming thresholds of energy and fuel spend intensity (Step 5 in Table 5). In both scopes, we observe an increasing RSQ value with increased levels of trimming. Both the mean and median AE significantly reduce as the trimming threshold increases, with median APE reaching 30% and 34% for Scope 1 and 2, respectively. To balance data quality with sample size, we apply a conservative 10% trimming, removing outliers while preserving diversity and representativeness.

**TABLE 5:** Performance metrics for Model 3 across different sample restrictions.

Metric		Unit	5% Trim	10% Trim	15% Trim	20% Trim	25% Trim
**Scope 1**							
Firms			50,290	39,704	30,217	21,962	15,140
Industries			46	44	41	36	34
RSQ (Fixed)			0.379	0.408	0.440	0.418	0.438
RSQ (Total)			0.855	0.879	0.898	0.921	0.932
Mean	AE	tCO_2_e	35.03	30.46	26.56	23.46	20.99
	APE	%	75.46%	62.71%	53.40%	46.30%	40.26%
Median	AE	tCO_2_e	7.72	6.80	6.02	5.33	4.71
	APE	%	45.29%	41.22%	37.36%	33.92%	30.43%
**Scope 2**							
Firms			104,272	92,714	81,094	69,477	57,903
Industries			54	54	54	52	52
RSQ (Fixed)			0.540	0.586	0.628	0.664	0.697
RSQ (Total)			0.662	0.717	0.761	0.798	0.832
Mean	AE	tCO_2_e	10,925	9476	8397	7547	6897
	APE	%	89.98%	73.60%	62.12%	53.22%	45.87%
Median	AE	tCO_2_e	2663	2364	2098	1848	1604
	APE	%	53.68%	48.52%	43.49%	38.61%	33.91%

Abbreviations: AE, absolute error; APE, absolute percentage error.

## DISCUSSION

### Key findings

New modeling approaches, such as the one detailed in this paper, offer a practical way forward to addressing emissions data gaps among SMEs. Here, we have provided a use case for utilizing SMEs' FTD as proxies for emission-generating activities, estimating the emissions for an otherwise unobserved subset of the business population. We then use this emissions data to develop new models that predict Scope 1 and 2 emissions.

We identify models that can achieve an RSQ of 0.89 for Scope 1 emissions and 0.72 for Scope 2 emissions. This translates to a reduction in error of over 50% when compared to the simple estimation techniques currently used under frameworks such as PCAF. This error reduction can be attributed to three main findings. First, we observe a consistent, strong, and significant relationship between *ln* turnover and our dependent variables (*ln* Scope 1 and 2 emissions) with a coefficient of 0.7 at the lowest. This log–log relationship implies that the relationship between the raw variables is not linear, as is assumed under simple estimation techniques. Second, we find that accounting for industry characteristics beyond basic emission intensity significantly improves model performance, particularly for Scope 1 emissions. We observe a model performance increase of nearly 10% by incorporating industry skew, underscoring the importance of additional factors currently overlooked. Finally, we find that the inclusion of detailed, firm-specific variables to try and explain heterogeneities beyond size leads to diminishing model performance gains, while significantly disadvantaging the practical application of the model. A pragmatic approach balances complexity with accuracy to enhance accessibility.

With these findings, this study supports the notion that regression analyses can provide an alternative method to estimating emission data in the absence of reported data (Goldhammer et al., 2017). By tailoring the model specifically to the SME context, we address a critical limitation in the current literature (Assael et al., 2023), ensuring inclusion of firms with the highest likelihood of emissions data gaps.

### Practical implications

Regulatory exclusions and firm size–specific barriers have kept SMEs largely absent from emissions reporting, limiting their engagement in improving sustainability and leaving their environmental impact poorly understood. The emission prediction models described here are made available in Supporting Information S6, enabling users to produce SME emission estimates from minimal inputs (namely turnover and industry), while improving on the accuracy of sector-level average estimates.

These models give SMEs a crucial starting point to both understand and reduce their environmental impact—an unmet need in the current landscape (BBB, 2024). When delivered through behaviorally informed design packages tailored to the specific barriers SMEs face, emissions estimates can help simplify the complex landscape of SME net-zero support. This approach tackles structural barriers like limited funding and expertise (Caldera et al., 2019; Menon & Ravi, 2021), while also allowing tailored support for common SME constraints, such as occupying rented premises (Mazhar et al., 2024).

The benefits of increased SME engagement through model availability drive the adoption of sustainability practices, help SMEs maintain competitiveness against larger firms, and remove obstacles to accessing external funding (Appiah-Kubi et al., 2024; Madrid-Guijarro & Duréndez, 2024). Additional impacts may be achieved when emission estimates are distributed by FIs with academic endorsement, leveraging established, trusted relationships.

With this paper, we also begin to build the argument for FTD as a viable option for emission estimation at scale, particularly among smaller actors. This is an argument developed in the household space by Trendl et al. (2023) and Wells et al. (2025), but one not yet applied in the corporate emission reporting environment. This approach enables automated emission insights for SMEs while allowing external parties to understand emissions drivers at scale. Integrating FTD-based emissions data into the PCAF framework for estimating portfolio-financed emissions could incentivize FIs to leverage their extensive data assets to develop methodologies that serve the public good.

### Limitations

While modeling approaches improve accessibility, key limitations exist. Some industries are excluded due to limited sample sizes or unreliable turnover estimates. The method is also less suitable for sectors like agriculture, where large proportions of emissions arise from processes not captured in FTD. As a result, full sectoral coverage is not achieved.

A full list of excluded SIC codes and the implications of their removal is provided in Table 7 of Supporting Information S1. To assess the impact of exclusions, we compare business population data (BEIS, 2022) against the prediction model coverage to identify SIC codes that have the highest exclusion impact. We find that the model covers over 71% of the UK SME population, with scope to expand this further if trade-offs in accuracy are acceptable. Of the excluded SMEs, 25% are excluded due to our initial exclusions, with an additional 4% excluded because of sample size within our restriction process. Of the excluded industries, most represent a minimal share of the UK's SME population. Notable exceptions include agriculture and service sectors such as insurance, real estate, legal, and accounting, which do contain SME populations of 5% and 16% of the SME population, respectively.

Additionally, FTD lacks the granularity of receipt-level analysis. Emission conversion factors are therefore applied at the merchant category level only (Trendl et al., 2023). This means transaction-based emission estimation relies on assumptions regarding energy mix, since many energy suppliers provide both natural gas and electricity, and petrol stations sell both diesel and petrol. Although these assumptions are informed by official industry surveys, they remain approximations. Related to this, Scope 2 emissions are estimated using location-based emission intensity factors. For SMEs on renewable energy tariffs, emissions are overestimated when looking from a market-based perspective. Our model is primarily designed to predict energy-use emissions rather than precisely reflect specific tariff choices, and in a publicly available version of the tool, users would have the option to toggle renewable tariff use on or off. This would both highlight the emission reduction opportunities of green tariffs and present a clear, actionable step for firms not yet on a renewable tariff.

This analysis reflects a 1-year snapshot, aligning with practical deployment. Future iterations of the model can be easily updated, with new FTD and emissions conversion factors. In addition, the model is developed with UK data; its applicability is therefore limited to non-cash-based, digital economies with comprehensive FTD. SME activity is often shaped by regional factors, and model development for other economies requires changes to conversion factors, currencies, grid intensities, and other differences. This presents an opportunity for collaborations between regional policymakers and financial institutions.

### Future Research Directions

While this paper focuses on Scope 1 and 2 emissions due to regulatory emphasis, a firm's complete footprint includes Scope 3 emissions. As a growing area of regulatory interest, Scope 3 presents a strong use case for FTD. Future research could explore applying FTD to estimate these emissions, potentially enabling broader reporting of this underrepresented category while reducing the effort and cost associated with measurement (Hettler & Graf-Vlachy, 2024). This may require integrating new data sources or methods to better capture supply chain emissions and provide a more complete SME profile.

A second area for further research is to compare transaction-based emission estimates for firms with alternative firm-level microdata. While Trendl et al. (2023) verify FTD as a credible alternative to survey-based household estimates, equivalent validation in the commercial sector remains unexplored. Addressing this gap would help clarify the current uncertainties around the validity of FTD emissions estimation. Potential comparable emissions data include self-disclosed reports such as those submitted under the SECR framework. However, these are constrained by limited sample sizes, business size thresholds, and the need for manual data collection. In this study, a manual compilation of SECR reports conducted for out-of-sample testing revealed several challenges with the dataset, which are discussed in detail in Supporting Information S4. Survey data, such as the Annual Business Survey (ONS, 2024b), offer broader sectoral and firm coverage, while avoiding the inconsistencies within self-reported methodologies. However, such sources present access barriers and do not permit one-to-one firm-level comparisons. Accountancy data also offer a promising avenue, with software providers increasingly integrating emissions profiling into their platforms (Sage Earth, 2025; Xero, 2025).

## CONCLUSION

This paper introduces a new statistical model for predicting Scope 1 and 2 emissions of SMEs using widely accessible information such as company turnover, industry classification, and sectoral characteristics from public accounts. By fitting the model to FTD data from over 100,000 UK SMEs, we capture key firm and industry-level relationships that enable it to outperform commonly used benchmarking approaches.

A key finding from this research is that a relatively simple model requiring only four variables, two publicly available industry variables and a firm's industry and annual turnover, can generate accurate and robust emission estimates while remaining practical for real-world application. These types of modeling solutions can address important gaps in emission reporting for SMEs, who lack the resources and time to dedicate to sustainability challenges. By simplifying access to more accurate emission profiles, we can increase engagement among SMEs and provide their stakeholders, including financial lenders and policymakers, with information for targeting sustainability strategies.

## ENDNOTE

^1^SECR disclosure requirements cover companies who meet at least two of the following criteria: (1) turnover of £36m or more; (2) balance sheet assets of £18m or more; (3) 250 employees or more. Companies consuming less than 40,000 kWh of energy per year are exempt.

## Supplementary Information

**Supporting Information S1**: This supporting information provides a comprehensive description of the sample selection process. It includes a table detailing the rationale and impact of each step involved in generating the final samples. Additionally, pre- and post-restriction distributions are presented to illustrate the correction of irregularities. A complete list of SIC codes is also provided, with excluded industries identified and the rationale alongside sample and population sizes of each SIC industry.

**Supporting Information S2**: This supporting information provides the utility adjustment factors for differing consumption levels of energy.

**Supporting Information S3**: This supporting information provides details on the calculation of key data required to estimate Scope 1 and 2 emissions from financial transactions. This includes the calculation of downstream emission factors, and subsequent sample summary statistics.

**Supporting Information S4**: This supporting information outlines the model's out-of-sample performance, evaluated against both FTD-based estimates and self-reported emissions.

**Supporting Information S5**: This supporting information provides supporting data for all figures presented in this paper.

**Supporting Information S6**: This supporting information directs the reader to the publicly available git repository, where the model described in this paper can be used to generate Scope 1 and 2 emission estimates.

## Data Availability

The data that support the findings of this study are available from Lloyds Banking Group, but restrictions apply to the availability of these data, which were used under license for the current study, and so are not publicly available. Data may be available from the authors upon reasonable request and with permission from LBG.
