# Supplementary material for: Bridging the SME reporting gap: A new model for predicting Scope 1 and 2 emissions
Source: J Ind Ecol. 2025 Sep 23;29(6):2197–213. doi: 10.1111/jiec.70106 (PMC13279493; doi:10.1111/jiec.70106)
Supplement: Supplementary file 1 — Supporting Information S1: This supporting information provides a comprehensive description of the sample selection process. It includes a table detailing the rationale and impact of each step involved in generating the final samples. Additionally, pre- and post-restriction distributions are presented to illustrate the correction of irregularities. A complete list of SIC codes is also provided, with excluded industries identified and the rationale alongside sample and population sizes of each SIC industry. [file 44498_2025_2906019_MOESM1_ESM.docx]

# Supporting Information S1 – Sample Selection Process

This document provides a comprehensive description of the sample selection process. It includes a table detailing the rationale and impact of each step involved in generating the final samples. Additionally, pre- and post-restriction distributions are presented to illustrate the correction of irregularities. A complete list of SIC codes is also provided, with excluded codes clearly identified along with the corresponding justification.

Table 6 outlines the restriction process required to procure our sample for Scope 1 and 2 emission estimation. Each step is provided with corresponding rationale and sample impact (number of firms and industries remaining in the sample). For steps 2, 3, and 4 which involve the creation of metrics used to identify representative data, we also provide pre- and post-distribution plots. This can be used to understand the challenges we intend to correct for, and output distributions.

Finally, in Table 7 we include a full list of SIC codes, with excluded industries identified and the rationale alongside sample and population sizes of each SIC, presenting the impact of industry exclusions. This relates to the rationale of step 1a, and is discussed further in Section 5.2.

| **Restriction Step** | | **Rationale** | **Impact** | | | |
| --- | --- | --- | --- | --- | --- | --- |
|  |  |  | **Scope 1** | | **Scope 2** | |
|  |  |  | **Firms** | **Industries** | **Firms** | **Industries** |
| 1a. | Eligible sample | Exclude firms that operate within flagged industries. | 424,764 | 60 | 424,764 | 60 |
| 1b. |  | Exclude firms outside defined SME revenue brackets^[[1]](#footnote-2)^. | 352,811 | 60 | 352,811 | 60 |
| 1c. |  | Exclude firms with zero scope related spend. | 97,957 | 54 | 166,169 | 55 |
| 2. | Minimum account activity | Exclude firms with dormant accounts, we set a minimum threshold for total transactions to 60 in 2021 (averaging 5 per month). | 97,608 | 54 | 161,845 | 55 |
| 3. | Account use metric | To identify and exclude firms with unusual bank account activity we divide the number of annual transactions made by firm turnover. We group firms by size and industry and remove the remove the upper and lower 10% of each distribution. | 77,771 | 52 | 129,171 | 55 |
| 4. | Energy and fuel spend to turnover | To remove outliers, caused by artificially low or high turnover levels,  we divide annual energy and fuel spend with turnover and exclude the top and bottom 5%. This step ensures turnover figures are representative, consider the firm’s energy and fuel spend. | 62,828 | 52 | 116,211 | 55 |
| 5. | Energy and fuel spend to total spend | Finally, we calculate an energy intensity metric, dividing energy and fuel spend by total spend. In this process, we identify firms with artificially high or low energy and fuel spend as a share of their total spend. We group firms by size and industry and remove the upper and lower 15% of each distribution. We conduct a sensitivity analysis to assess the impact of varying the sample restriction thresholds and present these results in Section 3.3. | 39,702 | 44 | 92,714 | 54 |

Table 6. Sample restriction steps and impact

**Pre- and Post- Distribution Plots**

Step 3 – Account Use Metric

After calculating the account usage metric (annual transaction count divided by turnover), we identify issues at both ends of the distribution. Firstly, we notice a substantial number of near-zero values, indicating that account usage is exceedingly low relative to firm size. Secondly, we observe a long right tail, which signifies the presence of firms with exceptionally high account usage relative to their size. To address these disparities, we group firms by industry and size, subsequently trimming the lower and upper 10%. This results in a refined distribution post-restriction.

Figure 4. Pre- and post- restriction plots for account usage


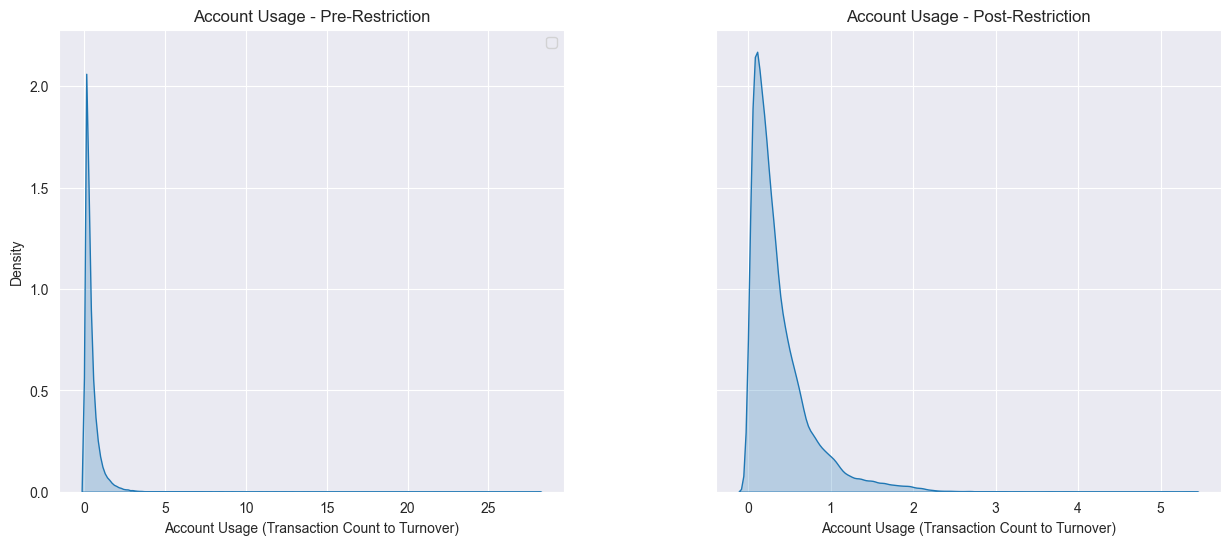

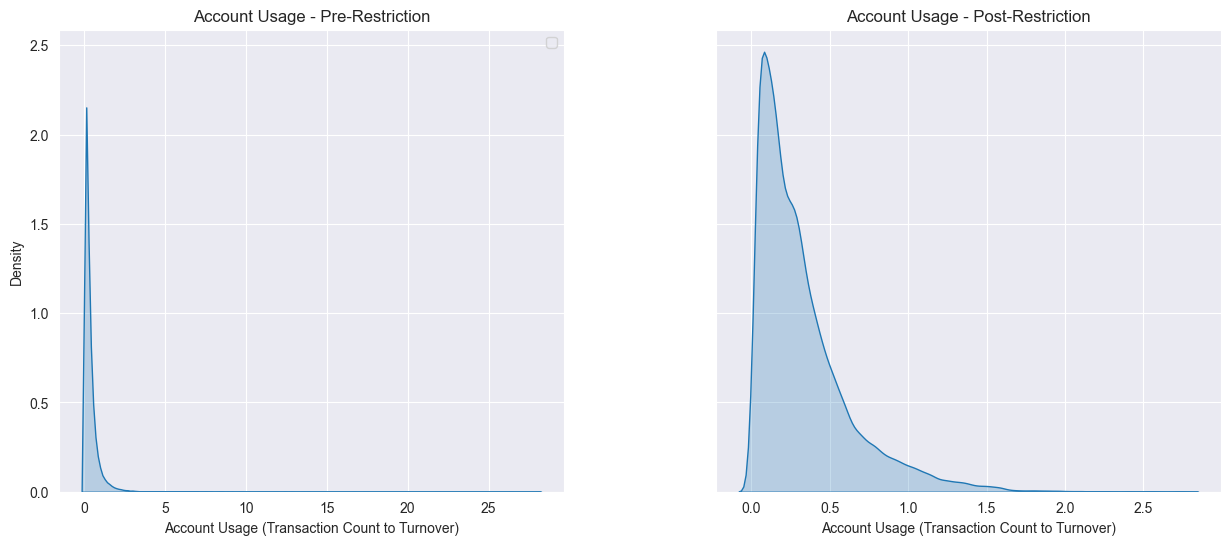


Scope 1

Scope 2

Step 4 – Energy and Fuel spend relative to Turnover

Step 4 – Energy and fuel spend to turnover

After calculating energy and fuel expenditures relative to turnover, we again identify issues at both ends of the distribution for each fuel type. As previously noted, a significant number of near-zero values indicate low spending in these categories relative to firm size. Additionally, we observe extremely long right tails, suggesting that some accounts exhibit exceptionally high spending in these areas relative to their size. Given that this step aims primarily to eliminate outliers resulting from artificially high or low observed turnover levels, we only group firms by industry before removing the lower and upper 5% of the distribution. Notably, we find that right tails persist to an extent, particularly in the fuel and gas categories. Upon further investigation, we determine that the firms within these extremes belong to a limited number of industries, leading us to conclude that fuel and gas consumption may indeed be higher in these specific sectors.

Figure 5. Pre- and post- restriction plots for energy and fuel spend to turnover


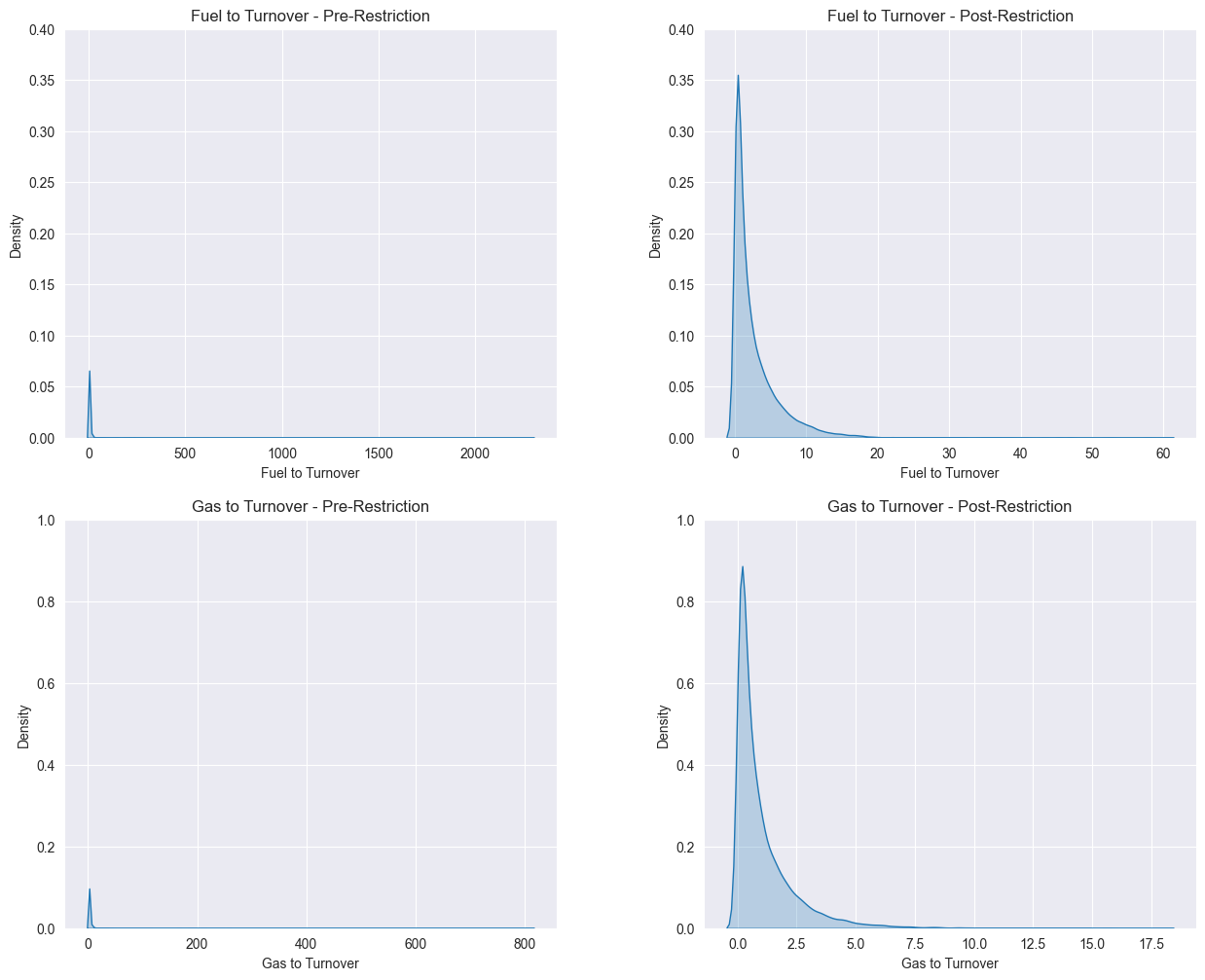

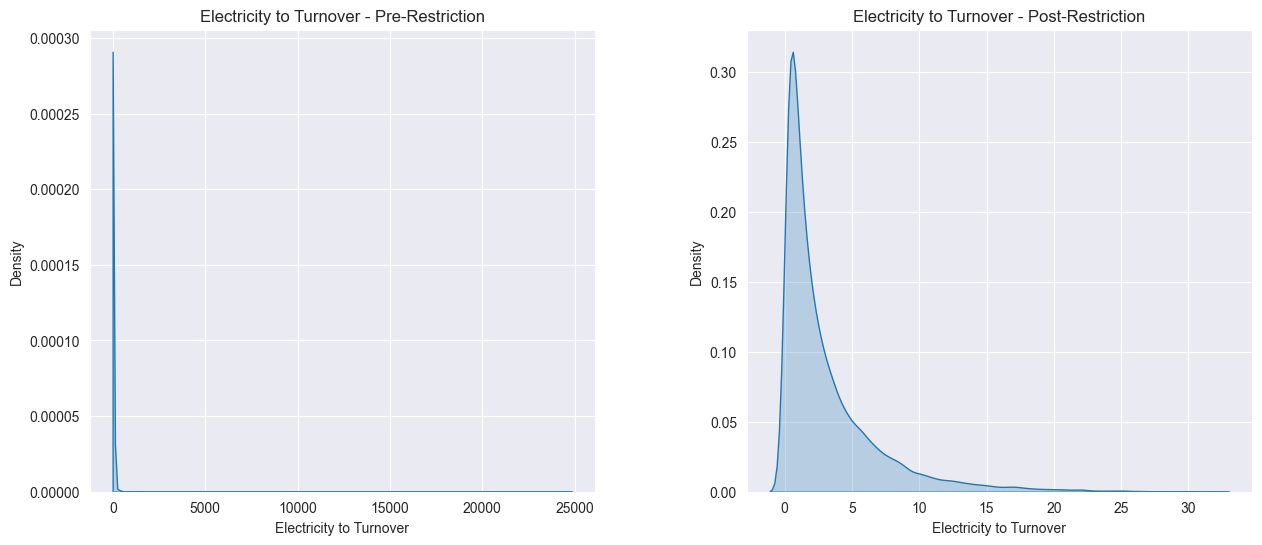


Step 5 – Energy and fuel spend to total spend

After resolving issues related to turnover and general account usage, we introduce a restriction to control for the proportion of total expenditure that firms allocate to energy and fuel. Prior to this restriction, we observe that many firms spend close to 0% of their total budget on energy and fuel, while others allocate more than 50%. We hypothesize that this variation stems from some firms maintaining dedicated accounts for energy and fuel, which may not always be captured in our data. To refine the sample, we group firms by industry and size, removing the lower and upper 15% of the distribution. As this step is more arbitrary, we report model performance for different levels of trimming, in Section 3.3.2.

Figure 6. Pre- and post- restriction plots for energy and fuel spend to total spend


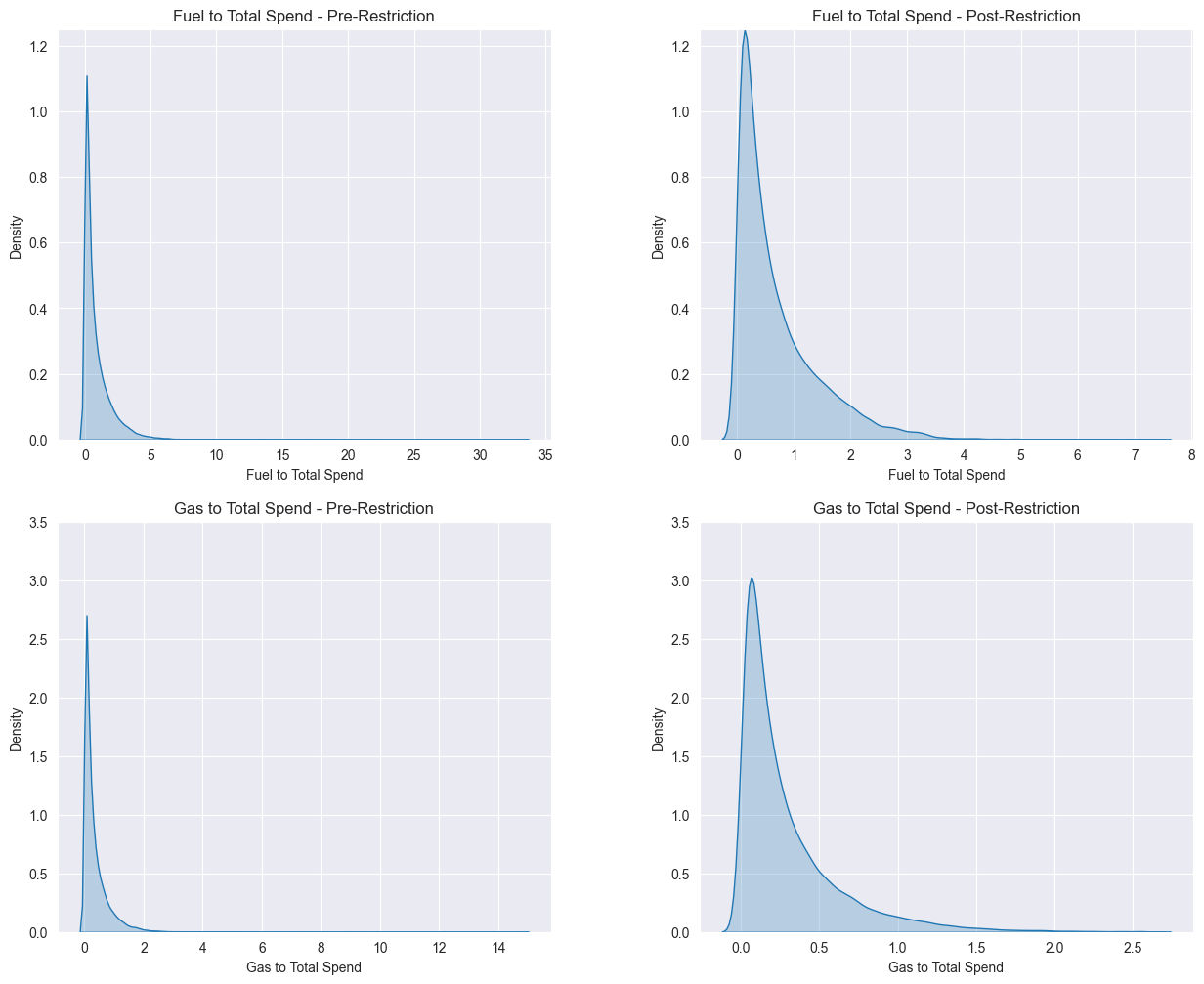

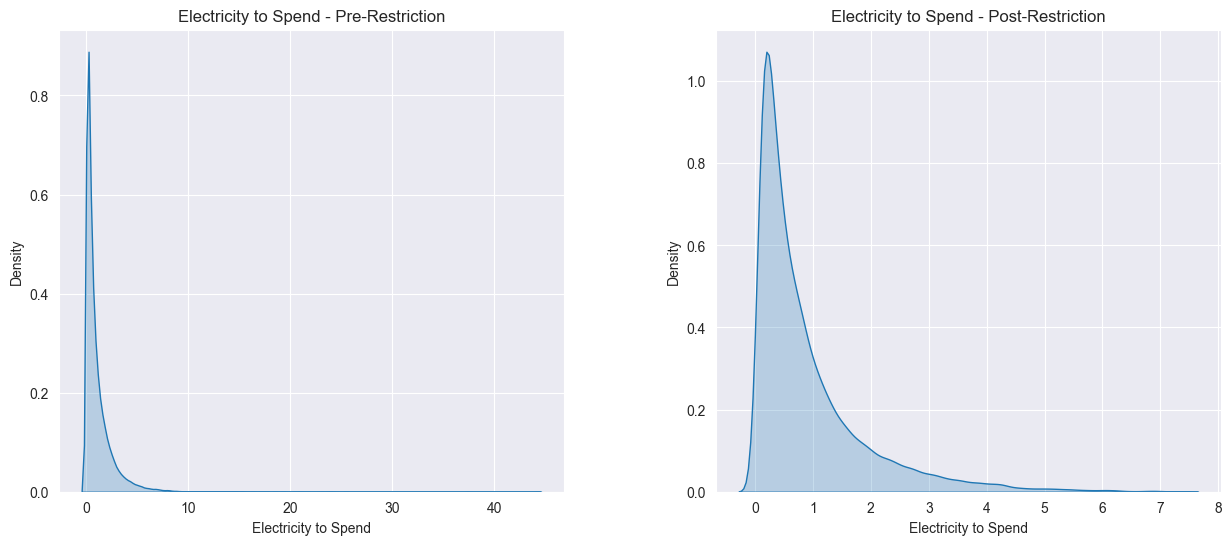


**Full list of SIC codes, with excluded industries identified and the rationale**

Table 7. SIC code sample and population sizes, exclusion stage and rationale

| **Section** | **Division** | **Description** | **Excluded before sample selection?** | **Excluded during sample selection?** | **Starting Sample Size** | **Final Sample** | | **UK Business Population** | | | **Notes** |
| --- | --- | --- | --- | --- | --- | --- | --- | --- | --- | --- | --- |
|  |  |  |  |  |  | **Scp 1** | **Scp 2** | **Total Firms** | **SMEs*** | **SMEs*** |  |
|  |  |  |  |  |  |  |  |  |  |  |  |
|  |  |  |  |  | ***#*** | ***#*** | ***#*** | ***#*** | ***#*** | ***%*** |  |
| A | 1 | Crop and animal production, hunting and related service activities | Y |  | - | - | - | 132,420 | 132,335 | *99.94* | Industry identified as high error due to limitations of transaction methodology |
| A | 2 | Forestry and logging |  |  | 726 | 79 | 129 | 4,440 | 4,440 | *100.00* |  |
| A | 3 | Fishing and aquaculture |  |  | 955 | 79 | 153 | 4,160 | 4,155 | *99.88* |  |
| B | 5 | Mining of coal and lignite | Y |  | - | - | - | 10 | 10 | *100.00* | Missing required industry data for regression input variables |
| B | 6 | Extraction of crude petroleum and natural gas |  | Y | - | - | - | 150 | 130 | *86.67* |  |
| B | 7 | Mining of metal ores | Y |  | - | - | - | - | - | *0.00* | Missing required industry data for regression input variables |
| B | 8 | Other mining and quarrying |  | Y | 177 | - | - | 705 | 695 | *98.58* |  |
| B | 9 | Mining support service activities |  | Y | 310 | - | - | 385 | 375 | *97.40* |  |
| C | 10 | Manufacture of food products |  |  | 1,551 | 227 | 470 | 8,945 | 8,715 | *97.43* |  |
| C | 11 | Manufacture of beverages |  |  | 449 | 66 | 139 | 2,730 | 2,700 | *98.90* |  |
| C | 12 | Manufacture of tobacco products | Y |  | - | - | - | 5 | 5 | *100.00* | Missing required industry data for regression input variables |
| C | 13 | Manufacture of textiles |  |  | 1,314 | 134 | 306 | 4,290 | 4,270 | *99.53* |  |
| C | 14 | Manufacture of wearing apparel |  | Y | 606 | - | 136 | 4,245 | 4,240 | *99.88* |  |
| C | 15 | Manufacture of leather and related products |  | Y | 157 | - | - | 630 | 625 | *99.21* |  |
| C | 16 | Manufacture of wood and of products of wood and cork, except furniture; manufacture of articles of straw and plaiting materials |  |  | 2,533 | 365 | 653 | 9,940 | 9,920 | *99.80* |  |
| C | 17 | Manufacture of paper and paper products |  | Y | 272 | - | 82 | 1,430 | 1,395 | *97.55* |  |
| C | 18 | Printing and reproduction of recorded media |  |  | 2,463 | 257 | 642 | 10,940 | 10,910 | *99.73* |  |
| C | 19 | Manufacture of coke and refined petroleum products |  | Y | - | - | - | 90 | 80 | *88.89* |  |
| C | 20 | Manufacture of chemicals and chemical products |  |  | 716 | 88 | 208 | 3,230 | 3,155 | *97.68* |  |
| C | 21 | Manufacture of basic pharmaceutical products and pharmaceutical preparations |  | Y | - | - | - | 695 | 645 | *92.81* |  |
| C | 22 | Manufacture of rubber and plastic products |  |  | 1,202 | 230 | 419 | 5,550 | 5,460 | *98.38* |  |
| C | 23 | Manufacture of other non-metallic mineral products |  |  | 1,239 | 180 | 338 | 3,810 | 3,765 | *98.82* |  |
| C | 24 | Manufacture of basic metals |  |  | 484 | 100 | 172 | 1,825 | 1,790 | *98.08* |  |
| C | 25 | Manufacture of fabricated metal products, except machinery and equipment |  |  | 8,011 | 1,382 | 2,345 | 27,805 | 27,730 | *99.73* |  |
| C | 26 | Manufacture of computer, electronic and optical products |  |  | 1,039 | 100 | 248 | 5,755 | 5,695 | *98.96* |  |
| C | 27 | Manufacture of electrical equipment |  |  | 2,349 | 299 | 564 | 3,090 | 3,040 | *98.38* |  |
| C | 28 | Manufacture of machinery and equipment nec |  |  | 2,195 | 411 | 712 | 7,460 | 7,370 | *98.79* |  |
| C | 29 | Manufacture of motor vehicles, trailers and semi-trailers |  |  | 1,015 | 141 | 275 | 3,495 | 3,405 | *97.42* |  |
| C | 30 | Manufacture of other transport equipment |  |  | 893 | 111 | 200 | 2,265 | 2,200 | *97.13* |  |
| C | 31 | Manufacture of furniture |  |  | 1,577 | 259 | 476 | 6,580 | 6,550 | *99.54* |  |
| C | 32 | Other manufacturing |  |  | 2,637 | 218 | 543 | 10,210 | 10,175 | *99.66* |  |
| C | 33 | Repair and installation of machinery and equipment |  |  | 342 | 50 | 100 | 15,085 | 15,030 | *99.64* |  |
| D | 35 | Electricity, gas, steam and air conditioning supply | Y |  | - | - | - | 5,840 | 5,795 | *99.23* | Industry identified as high error due to limitations of transaction methodology |
| E | 36 | Water collection, treatment and supply | Y |  | - | - | - | 105 | 85 | *80.95* | Industry identified as high error due to limitations of transaction methodology |
| E | 37 | Sewerage | Y |  | - | - | - | 1,105 | 1,105 | *100.00* | Industry identified as high error due to limitations of transaction methodology |
| E | 38 | Waste collection, treatment and disposal activities; materials recovery | Y |  | - | - | - | 5,915 | 5,865 | *99.15* | Industry identified as high error due to limitations of transaction methodology |
| E | 39 | Remediation activities and other waste management services | Y |  | - | - | - | 1,150 | 1,145 | *99.57* | Industry identified as high error due to limitations of transaction methodology |
| F | 41 | Construction of buildings |  |  | 9,365 | 845 | 2,080 | 111,955 | 111,845 | *99.90* |  |
| F | 42 | Civil engineering |  |  | 17,195 | 2,566 | 4,091 | 25,105 | 25,025 | *99.68* |  |
| F | 43 | Specialised construction activities |  |  | 62,387 | 8,597 | 13,901 | 222,640 | 222,530 | *99.95* |  |
| G | 45 | Wholesale and retail trade and repair of motor vehicles and motorcycles |  |  | 19,402 | 3,077 | 5,784 | 78,995 | 78,790 | *99.74* |  |
| G | 46 | Wholesale trade, except of motor vehicles and motorcycles |  |  | 10,937 | 1,553 | 3,079 | 106,735 | 106,250 | *99.55* |  |
| G | 47 | Retail trade, except of motor vehicles and motorcycles |  |  | 43,590 | 4,595 | 12,490 | 220,685 | 220,205 | *99.78* |  |
| H | 49 | Land transport and transport via pipelines | Y |  | - | - | - | 79,450 | 79,270 | *99.77* | Industry identified as high error due to limitations of transaction methodology |
| H | 50 | Water transport |  | Y | 248 | - | - | 1,360 | 1,350 | *99.26* |  |
| H | 51 | Air transport |  | Y | - | - | - | 1,045 | 1,025 | *98.09* |  |
| H | 52 | Warehousing and support activities for transportation |  |  | 2,539 | 252 | 526 | 17,380 | 17,210 | *99.02* |  |
| H | 53 | Postal and courier activities |  | Y | - | - | - | 39,170 | 39,140 | *99.92* |  |
| I | 55 | Accommodation |  |  | 6,815 | 1,117 | 2,616 | 18,665 | 18,485 | *99.04* |  |
| I | 56 | Food and beverage service activities |  |  | 24,292 | 3,426 | 9,800 | 148,350 | 147,885 | *99.69* |  |
| J | 58 | Publishing activities |  | Y | 2,291 | - | 202 | 12,075 | 12,015 | *99.50* |  |
| J | 59 | Motion picture, video and television programme production, sound recording and music publishing activities |  |  | 2,216 | 61 | 178 | 29,320 | 29,280 | *99.86* |  |
| J | 60 | Programming and broadcasting activities |  | Y | 879 | - | 65 | 2,035 | 2,020 | *99.26* |  |
| J | 61 | Telecommunications |  | Y | 907 | - | 141 | 8,440 | 8,385 | *99.35* |  |
| J | 62 | Computer programming, consultancy and related activities |  |  | 12,766 | 306 | 1,031 | 151,945 | 151,720 | *99.85* |  |
| J | 63 | Information service activities |  | Y | 707 | - | 62 | 9,150 | 9,115 | *99.62* |  |
| K | 64 | Financial service activities, except insurance and pension funding | Y |  | - | - | - | 19,645 | 19,505 | *99.29* | Industry identified as high error due to limitations of transaction methodology |
| K | 65 | Insurance, reinsurance and pension funding, except compulsory social security | Y |  | - | - | - | 7,355 | 7,300 | *99.25* | Industry identified as high error due to limitations of transaction methodology |
| K | 66 | Activities auxiliary to financial services and insurance activities | Y |  | - | - | - | 34,315 | 34,115 | *99.42* | Industry identified as high error due to limitations of transaction methodology |
| L | 68 | Real estate activities | Y |  | - | - | - | 105,370 | 105,145 | *99.79* | Industry identified as high error due to limitations of transaction methodology |
| M | 69 | Legal and accounting activities | Y |  | - | - | - | 75,620 | 75,355 | *99.65* | Industry identified as high error due to limitations of transaction methodology |
| M | 70 | Activities of head offices; management consultancy activities | Y |  | - | - | - | 173,650 | 173,520 | *99.93* | Industry identified as high error due to limitations of transaction methodology |
| M | 71 | Architectural and engineering activities; technical testing and analysis |  |  | 11,466 | 683 | 1,714 | 93,470 | 93,310 | *99.83* |  |
| M | 72 | Scientific research and development |  | Y | 1,192 | - | 132 | 5,785 | 5,695 | *98.44* |  |
| M | 73 | Advertising and market research |  |  | 3,718 | 138 | 413 | 23,275 | 23,180 | *99.59* |  |
| M | 74 | Other professional, scientific and technical activities |  |  | 6,939 | 335 | 809 | 77,290 | 77,270 | *99.97* |  |
| M | 75 | Veterinary activities |  | Y | 942 | - | 261 | 3,890 | 3,880 | *99.74* |  |
| N | 77 | Rental and leasing activities | Y |  | - | - | - | 18,155 | 18,090 | *99.64* | Industry identified as high error due to limitations of transaction methodology |
| N | 78 | Employment activities |  |  | 4,251 | 195 | 582 | 30,615 | 30,155 | *98.50* |  |
| N | 79 | Travel agency, tour operator and other reservation service and related activities |  | Y | - | - | - | 8,670 | 8,630 | *99.54* |  |
| N | 80 | Security and investigation activities |  | Y | 154 | - | - | 9,910 | 9,835 | *99.24* |  |
| N | 81 | Services to buildings and landscape activities |  |  | 12,575 | 1,012 | 1,672 | 46,525 | 46,225 | *99.36* |  |
| N | 82 | Office administrative, office support and other business support activities |  |  | 10,662 | 522 | 1,310 | 116,340 | 116,160 | *99.85* |  |
| O | 84 | Public administration and defence; compulsory social security |  | Y | 209 | - | - | 7,695 | 7,295 | *94.80* |  |
| P | 85 | Education |  |  | 18,276 | 913 | 3,061 | 45,490 | 43,935 | *96.58* |  |
| Q | 86 | Human health activities |  |  | 21,558 | 1,019 | 4,648 | 57,620 | 57,170 | *99.22* |  |
| Q | 87 | Residential care activities |  |  | 1,384 | 187 | 483 | 10,825 | 10,420 | *96.26* |  |
| Q | 88 | Social work activities without accommodation |  |  | 25,189 | 605 | 2,655 | 36,110 | 35,790 | *99.11* |  |
| R | 90 | Creative, arts and entertainment activities |  |  | 6,933 | 246 | 686 | 29,955 | 29,925 | *99.90* |  |
| R | 91 | Libraries, archives, museums and other cultural activities |  | Y | 670 | - | 153 | 1,830 | 1,780 | *97.27* |  |
| R | 92 | Gambling and betting activities |  | Y | - | - | - | 975 | 940 | *96.41* |  |
| R | 93 | Sports activities and amusement and recreation activities |  |  | 21,910 | 951 | 2,618 | 35,495 | 35,265 | *99.35* |  |
| S | 94 | Activities of membership organisations |  | Y | 4,391 | - | 343 | 22,340 | 22,265 | *99.66* |  |
| S | 95 | Repair of computers and personal and household goods |  | Y | - | - | - | 9,665 | 9,650 | *99.84* |  |
| S | 96 | Other personal service activities |  |  | 20,597 | 1,727 | 5,818 | 76,275 | 76,245 | *99.96* |  |
| T | 97 | Activities of households as employers of domestic personnel | Y |  | - | - | - | - | - | *0.00* | Missing required industry data for regression input variables |
| T | 98 | Undifferentiated goods- and services-producing activities of private households for own use | Y |  | - | - | - | 5 | 5 | *100.00* | Missing required industry data for regression input variables |
| U | 99 | Activities of extraterritorial organisations and bodies | Y |  | - | - | - | - | - | *0.00* | Missing required industry data for regression input variables |
|  |  |  |  |  |  |  |  |  |  |  |  |
| **Included Firms** | | | **2,095,955** | **2,703,915** | **424,764** | **39,704** | **92,714** | **2,765,145** | **2,754,605** |  |  |
| ***Number of SIC Codes*** | | | ***21*** | ***23*** | ***60*** | ***44*** | ***54*** | ***85*** | ***85*** |  |  |
| ***Excluded*** | | | ***25.3%*** | ***4.1%*** |  |  |  |  |  |  |  |
| ***Captured*** | | | ***74.7%*** | ***95.9%*** | ***74%*** | ***71%*** | ***73%*** |  |  |  |  |

1. The Companies Act 2006 defines a large company as those companies operating with over 250 staff, and either an annual turnover exceeding £36m or its balance sheet exceeding £18 (Companies Act, 2006). Thus, in terms of turnover, we include firms operating with less than £36m annual turnover. Firms with an annual turnover below £10,000 are not deemed to be of sufficient size for inclusion in our analysis. [↑](#footnote-ref-2)
