# Supplementary material for: Bridging the SME reporting gap: A new model for predicting Scope 1 and 2 emissions
Source: J Ind Ecol. 2025 Sep 23;29(6):2197–213. doi: 10.1111/jiec.70106 (PMC13279493; doi:10.1111/jiec.70106)
Supplement: Supplementary file 2 — Supporting Information S2: This supporting information provides the utility adjustment factors for differing consumption levels of energy. [file 44498_2025_2906019_MOESM2_ESM.docx]

# Supporting Information S2 – Utility adjustment factors

This supporting information provides the calculation for the splitting of spend on "Energy and Utilities" into Scope 1 and Scope 2 elements, as well as provides the utility adjustment factors for differing consumption levels of energy.

**Splitting "Energy and Utilities" spend**

As many UK utility firms are known to provide electricity, gas, and heating oil to consumers, it is a necessary step to disaggregate spending into Scope 1 and Scope 2. To do this, we source external data which signals the expected split between spend on electricity and other fuels in the UK.

We find that national fuel consumption data is unsuitable, as it is published in physical units, and we require monetary values. Suitable monetary sources include supply and use tables or other survey-based indicators.

While the monetary data from supply and use tables is valid, it exhibits the limitation of requiring the same split percentage across all industries. A more detailed option comes from an ONS dataset on business energy spending intensity, which draws on two otherwise confidential sources: the Annual Business Survey (2019) and the Annual Purchases Survey (2018). This dataset includes the table “Energy Intensity by Industry Section and Energy Type,” which provides percentage breakdowns of energy spending Despite being based on 2018 data, we consider this the better option, as it allows for industry-specific variation. Table 8 below reproduces the original data and includes the subsequent calculation of industry-level splits.

To estimate the split between Scope 1 and Scope 2, we first sum the electricity and gas columns, then calculate the contribution of each fuel type. We exclude the "other" category, as supporting source documentation indicates it primarily consists of petroleum products, which are unlikely to be sold by utility companies.

For SIC sections G and I, the gas and other categories are reported as a combined figure due to confidentiality restrictions. To estimate gas intensity for these industries, we calculate the average gas and other intensities across all industries (excluding section D). We then determine the typical gas-to-other ratio based on these averages and apply that ratio to the combined figure to impute the gas intensity.

Table 8. Energy & Utilities split ratio calculation (Data highlighted grey represents raw data from source, whilst unhighlighted cells show our calculations).

|  |  | **Energy intensity by energy type** | | | | | |  | **Suppressed Value Assumption** | |  | **Utility Spend Split** | |
| --- | --- | --- | --- | --- | --- | --- | --- | --- | --- | --- | --- | --- | --- |
| **SIC Section** |  | **Electricity** | **Petrol/diesel** | **Natural gas** | **Other** | **Suppressed** | **Total** |  | **Gas Share** | **Utility Spend** |  | **Elec Share** | **Gas Share** |
|  |  | ***%*** | ***%*** | ***%*** | ***%*** | ***%*** | ***%*** |  | ***%*** | ***%*** |  | ***%*** | ***%*** |
| A |  | 1.2 | 10.0 | 0.1 | 0.6 | 0.0 | 11.9 |  |  | 1.3 |  | 94 | 6 |
| B |  | 2.1 | 3.3 | 1.3 | 1.2 | 0.0 | 7.9 |  |  | 3.4 |  | 61 | 39 |
| C |  | 1.8 | 0.7 | 0.8 | 9.4 | 0.0 | 12.7 |  |  | 2.6 |  | 69 | 31 |
| D |  | 33.5 | 0.3 | 20.8 | 1.7 | 0.0 | 56.3 |  |  | 54.3 |  | 62 | 38 |
| E |  | 6.3 | 5.3 | 0.4 | 0.1 | 0.0 | 12.2 |  |  | 6.8 |  | 94 | 6 |
| F |  | 0.4 | 2.2 | 0.1 | 0.1 | 0.0 | 2.8 |  |  | 0.5 |  | 78 | 22 |
| G |  | 2.5 | 3.5 |  |  | 3.7 | 9.7 |  | 1.6 | 4.0 |  | 62 | 38 |
| H |  | 1.5 | 14.0 | 0.2 | 8.8 | 0.0 | 24.5 |  |  | 1.7 |  | 91 | 9 |
| I |  | 4.6 | 4.9 |  |  | 4.7 | 14.2 |  | 1.9 | 6.5 |  | 71 | 29 |
| J |  | 2.1 | 0.4 | 0.1 | 0.0 | 0.0 | 2.6 |  |  | 2.2 |  | 96 | 4 |
| K |  | 0.8 | 0.2 | 0.1 | 0.0 | 0.0 | 1.0 |  |  | 0.9 |  | 92 | 8 |
| L |  | 4.1 | 1.2 | 0.8 | 0.1 | 0.0 | 6.2 |  |  | 4.9 |  | 83 | 17 |
| M |  | 0.9 | 1.1 | 0.2 | 0.1 | 0.0 | 2.3 |  |  | 1.1 |  | 84 | 16 |
| N |  | 1.4 | 3.5 | 0.4 | 0.4 | 0.0 | 5.7 |  |  | 1.9 |  | 77 | 23 |
| P |  | 2.5 | 1.7 | 0.9 | 0.2 | 0.0 | 5.3 |  |  | 3.4 |  | 73 | 27 |
| Q |  | 3.6 | 1.3 | 2.0 | 0.1 | 0.0 | 7.0 |  |  | 5.6 |  | 65 | 35 |
| R |  | 3.8 | 0.9 | 0.9 | 0.1 | 0.0 | 5.8 |  |  | 4.8 |  | 81 | 19 |
| S |  | 2.9 | 4.0 | 0.7 | 0.1 | 0.0 | 7.7 |  |  | 3.6 |  | 80 | 20 |
|  |  |  |  |  |  |  |  |  |  |  |  |  |  |
| *Mean (Excl D, G. I)* | | *2.27* | *2.37* | *0.59* | *0.84* |  | *6* |  |  |  |  | 78 | 22 |
|  |  |  |  |  |  |  |  |  |  |  |  |  |  |
|  |  |  |  | *41* | *59* |  |  |  |  |  |  |  |  |

**Utility Adjustment factors**

Transactions made with energy companies require additional adjustment steps, to account for discounts given to consumers of high energy spend. Businesses receive a cheaper unit price for energy as energy spend increases. As a result, we adjust spend upwards for higher consumption levels to reflect actual consumption levels (and thus emissions) – accounting for the lower unit price. We also adjust spend downwards for lower consumption levels to reflect the above average unit price charged to smaller consumers.

Table 9. Annual utility spend floors and the corresponding adjustment factors to apply.

| **Annual Utility Spend Floor** | **Utility Adjustment Factor** |
| --- | --- |
| £0 | 0.77 |
| £4,777 | 0.83 |
| £26,213 | 0.96 |
| £115,690 | 0.98 |
| £264,997 | 0.97 |
| £420,410 | 1.04 |
| £2,161,288 | 1.11 |
| £4,138,796 | 1.12 |
| £14,239,548 | 1.14 |
| £22,533,284 | 1.12 |
| £31,264,609 | 1.10 |
