# Supplementary material for: Bridging the SME reporting gap: A new model for predicting Scope 1 and 2 emissions
Source: J Ind Ecol. 2025 Sep 23;29(6):2197–213. doi: 10.1111/jiec.70106 (PMC13279493; doi:10.1111/jiec.70106)
Supplement: Supplementary file 3 — Supporting Information S3: This supporting information provides details on the calculation of key data required to estimate Scope 1 and 2 emissions from financial transactions. This includes the calculation of downstream emission factors, and subsequent sample summary statistics. [file 44498_2025_2906019_MOESM3_ESM.docx]

# Supporting Information S3 – Direct emission factors and subsequent sample summary statistics

This supporting information provides details on the calculation of key data required to estimate Scope 1 and 2 emissions from financial transactions. This includes the calculation of downstream emission factors, and subsequent sample summary statistics.

Table 9. Calculation of direct emission conversion factors

|  | **Scope 1** | | | | **Scope 2** |
| --- | --- | --- | --- | --- | --- |
|  | **Petrol** | **Diesel** | **Gas** | **Oil** | **Electricity** |
| Emissions per Unit | 2.19 | 2.51 | 0.18 | 2.54 | 0.21 |
| Average Unit Price (£) | 1.31 | 1.35 | 0.03 | 0.43 | 0.15 |
| Emissions per Pound | 1.67 | 1.86 | 5.96 | 5.94 | 1.41 |
| Consumption Split | 68% | 32% |  |  |  |
| **Conversion Factors** | **1.802** | | **5.949** | | **1.408** |

Table 10. Summary statistics by revenue bracket of Scope 1 and 2 emission estimates (kg CO_2_e)

|  | **Annual Revenue Brackets** | | | | | |
| --- | --- | --- | --- | --- | --- | --- |
|  | **< 50k** | **50 - 100k** | **100 - 250k** | **250 - 500k** | **500k - 1M** | **> 1M** |
| **Scope 1** |  |  |  |  |  |  |
| Mean | 5,193 | 9,973 | 15,226 | 28,382 | 49,200 | 221,143 |
| 25th Percentile | 2,494 | 4,921 | 6,970 | 13,079 | 21,112 | 59,473 |
| Median | 4,166 | 8,081 | 11,882 | 22,484 | 39,631 | 122,732 |
| 75th Percentile | 6,659 | 12,790 | 19,726 | 38,163 | 66,507 | 256,564 |
| Min | 235 | 576 | 542 | 707 | 869 | 763 |
| Max | 36,037 | 69,117 | 231,335 | 244,979 | 422,173 | 9,051,760 |
| Count | 5,159 | 6,622 | 8,355 | 6,302 | 5,383 | 7,881 |
|  |  |  |  |  |  |  |
| **Scope 2** |  |  |  |  |  |  |
| Mean | 2,148 | 3,748 | 6,909 | 11,423 | 17,840 | 62,589 |
| 25th Percentile | 741 | 1,128 | 1,905 | 3,488 | 5,591 | 13,106 |
| Median | 1,555 | 2,528 | 4,154 | 7,150 | 11,138 | 27,451 |
| 75th Percentile | 2,771 | 4,635 | 8,811 | 15,335 | 22,181 | 58,661 |
| Min | 38 | 101 | 130 | 340 | 588 | 885 |
| Max | 18,472 | 35,958 | 62,804 | 129,489 | 242,181 | 2,650,815 |
| Count | 14,238 | 15,680 | 20,114 | 14,487 | 11,239 | 16,956 |
