# Supplementary material for: Bridging the SME reporting gap: A new model for predicting Scope 1 and 2 emissions
Source: J Ind Ecol. 2025 Sep 23;29(6):2197–213. doi: 10.1111/jiec.70106 (PMC13279493; doi:10.1111/jiec.70106)
Supplement: Supplementary file 4 — Supporting Information S4: This supporting information outlines the model's out-of-sample performance, evaluated against both FTD-based estimates and self-reported emissions. [file 44498_2025_2906019_MOESM4_ESM.docx]

# Supporting Information S4 – Out of Sample Performance

This supporting information provides corresponding plots for the out of sample performance demonstrated in Section 4.2.2. We then provide out-of-sample performance metrics and plots for a small sample of self-reported emissions data (SECR).

**5-Fold Test and Train Plot**

We visualise our out-of-sample test and train results presented in Table 4 with Figure 7. Here we take a sample of 1,000 firms from the last test sample dataset, and plot predicted values against FTD emissions.


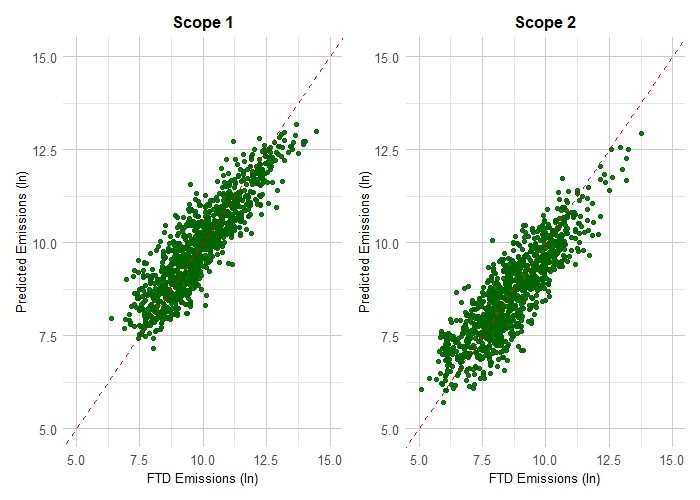
Figure 7 - Model predictions against out-of-sample FTD emission estimates. Underlying data for this figure are available in Table 6 within Supporting Information S5.

**SECR Out-of-sample Testing**

Further out-of-sample testing is conducted by comparing emissions predicted by our model against emissions self-reported by firms. To do this, we manually sourced and tabulated SECR reports for a sample of 50 firms via the Companies House website. For each firm, we input the reported turnover and SIC code into our model to generate estimates for Scope 1 and 2 emissions. These estimates are then compared to the corresponding self-reported values. Table 11 presents the summary results, with individual firm-level comparisons visualised in Figure 8. Following this, we provide our interpretation of these metrics, along with a detailed discussion of the limitations and key findings arising from the compilation of SECR data.

Table 11. Performance Metrics for Model 3 Test and Train Iterations

|  | | **Unit** | **Scope 1** | **Scope 2** |
| --- | --- | --- | --- | --- |
| **RSQ** | |  | 0.218 | 0.099 |
| **Mean** | **AE** | *t CO2e* | 714.48 | 704.61 |
|  | **APE** | *%* | 33.75 | 193.89 |
| **Median** | **AE** | *t CO2e* | 355.46 | 551.23 |
|  | **APE** | *%* | 25.32 | 65.08 |
|  | |  |  |  |

Figure 8 - Model predictions against out of sample FTD emission estimates. Underlying data for this figure are available in Table 7 within Supporting Information S5


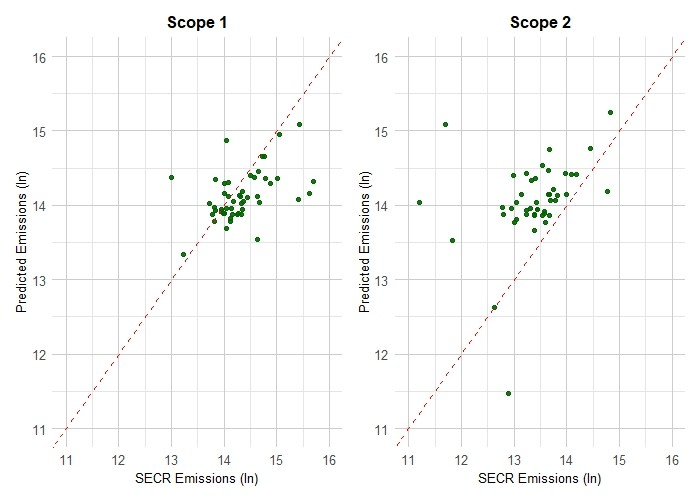


The Table 11 and Figure 8 reveal mixed results when applying our prediction model to SECR-reporting firms. While the majority of predicted values fall close to reported figures, there are notable outliers in both Scope 1 and Scope 2 emissions. These instances appear to be more extreme amongst Scope 2 predictions. When assessing the median AE and APE of Scope 1 predictions, we observe error levels consistent with error observed on out-of-sample FTD emissions, with a median APE of 25.32%. The median APE for Scope 2 is higher, at 65.08%. However, when assessed using mean AE and APE, the model exhibits higher error levels. This is also reflected in the low RSQ scores of 0.2 and 0.1 for Scope 1 and Scope 2, respectively.

Figure 8 also highlights a systematic overestimation of Scope 2 emissions for larger firms. This is consistent with the expectation that energy-use intensity tends to decline with firm size, driven by lower unit energy prices, economies of scale, and disproportionate increases in turnover beyond certain thresholds. Additionally, our model estimates Scope 2 emissions using location-based intensity factors, whereas individual firms often report using a market-based approach that accounts for renewable energy procurement, further contributing to estimate differences.

As previously mentioned, there are important limitations associated with these comparisons, which contribute to uncertainty regarding results. We identify three primary sources of uncertainty in this comparison:

First, and most critically, the SECR firms are substantially larger than those in our model sample. In the SECR sample, annual turnover ranges from £38 million to £211 million. By contrast, our model is trained exclusively on SMEs with turnover below £36 million, 80% of which fall under £1 million. As previously noted, larger firms may differ from SMEs within the same industry in terms of operational complexity, organisational behaviour, and emissions intensity. These differences highlight the need for an SME-specific approach to emissions estimation and contribute to the observed discrepancies in model performance.

Second, the reporting periods covered by SECR submissions do not always align precisely with our model’s reference year. Although we restrict our analysis to reports with a start or end date within 2021, company-specific reporting cycles vary and exact temporal alignment cannot be ensured.

Finally, we encountered numerous errors, inconsistencies, and omissions in the SECR reports themselves, making the dataset both difficult to compile and challenging to analyse. A key concern is the lack of consistency in the emissions conversion factors used: firms apply a wide range of factors, some from the relevant year, others from several years prior. Additional issues include unit conversion errors, such as confusion between MWh and kWh or between kg CO₂e and t CO₂e. This variability introduces further uncertainty into the comparison.
