# Supplementary material for: Bridging the SME reporting gap: A new model for predicting Scope 1 and 2 emissions
Source: J Ind Ecol. 2025 Sep 23;29(6):2197–213. doi: 10.1111/jiec.70106 (PMC13279493; doi:10.1111/jiec.70106)

# Supporting Information S6 – Accessing the model

To access the model described in this paper, the interested reader is directed to:

<https://david-leake.github.io/carbonpredict/>

This public repository allows external users to generate emission predictions for eligible SMEs using the model developed and tested in our paper. While the package is not yet available on CRAN, users can clone the repository and install it locally by following the instructions in the README file. Once the package is published on CRAN, it will be installable via install.packages("carbonpredict").

Example function use:


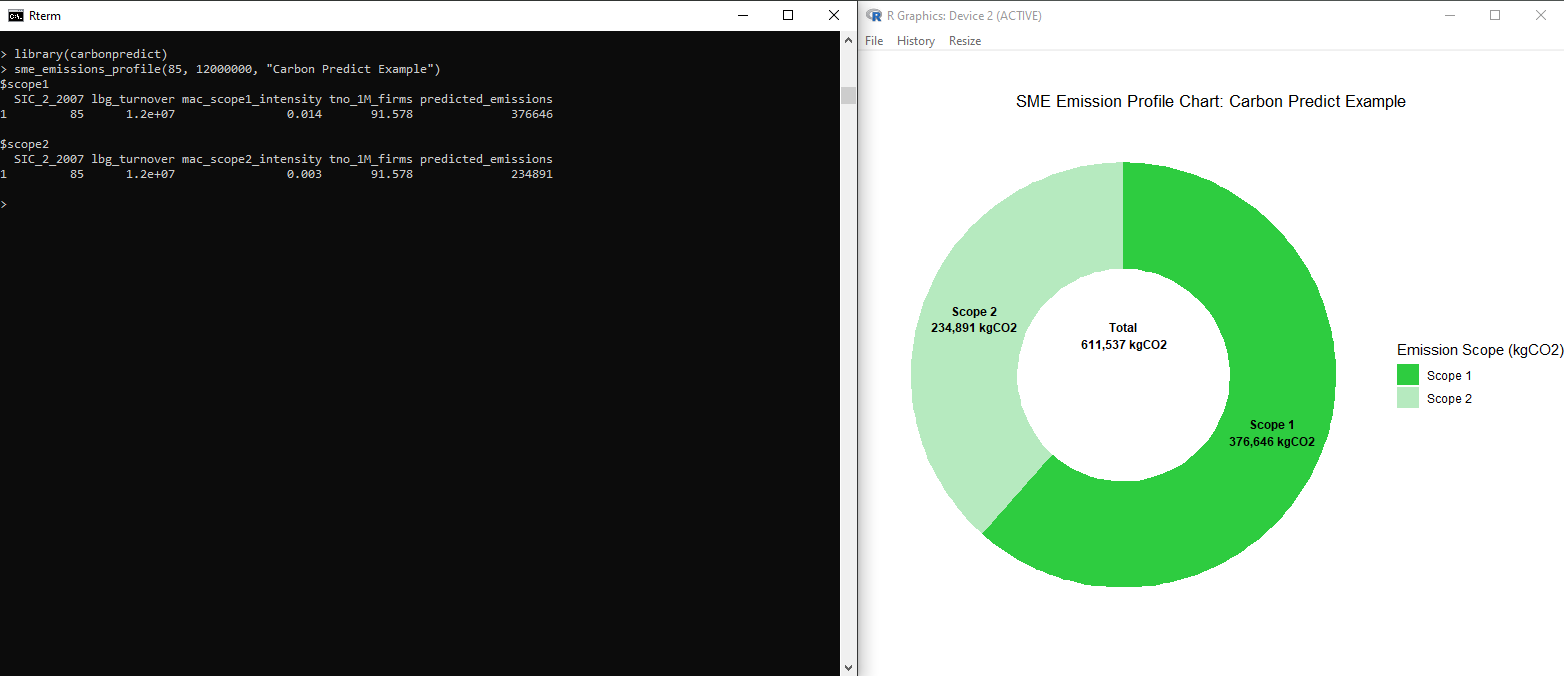

Supplement: Supplementary file 6 — Supporting Information S6: This supporting information directs the reader to the publicly available git repository, where the model described in this paper can be used to generate Scope 1 and 2 emission estimates. [file 44498_2025_2906019_MOESM6_ESM.docx]
